# Supplementary material for: Evaluating the Integrated Disease Surveillance and Response system in Sidama Region, Ethiopia: A systems evaluation
Source: PLoS One. 2026 May 21;21(5):e0349856. doi: 10.1371/journal.pone.0349856 (PMC13193418; doi:10.1371/journal.pone.0349856)
Supplement: S1 File — Questionnaire used to evaluate the Integrated Disease Surveillance and Response (IDSR) system at regional, district, health facility, health post, and community levels in Sidama Region, Ethiopia (September 2022). (PDF) [file pone.0349856.s001.pdf]

1. Annex 1: Study information sheets, consent forms and Questionnaires.

QUESTIONNAIRE FOR EVALUATION OF COMPRHENSIVE SURVEILLANCE SYSTEM IN REGIONAL, DISTRICT, HEALTH FACILITY, HEALTH POSTS & COMMUNITY SURVEILLANCE FOCAL POINTS, IN SIDAMA REGIONAL STATE, ETHIOPIA, SEPTEMBER-2022

Study information sheet:

My name is _____________ _______________. I belong to the research team studying the status of comprehensive public health surveillance system in the selected region, districts, health facilities, health posts and community surveillance focal in Sidama regional state. The study is being conducted by a [please express your self- Name, Status, Organization]

The objective of this study is to determine the status of the surveillance system by assessing, its core & supportive functions and the attributes. I kindly ask you to participate in this study and give me genuine answers for my queries. Your participation in this study is greatly helpful in identifying the status and factors in this area. The interview will take about 60 minutes and some supportive documents might be checked to support our observations. Your name will only be used for referencing and will never be used or mentioned in the report. You will not get payment because of your participation in this study and will not lose any too.

All information given by you will be kept confidential and no one except the research team members will have access to the information. Your participation is completely voluntary and you are not obligated to answer any question you are not willing to respond. If you feel any discomfort with the question, it is your right to drop it at any time you want. You may even decide not to engage in this study from the very beginning. I hope I have clarified the purposes of the study. If you have any question, you can ask me now or you may ask the principal investigator, Sileshi Demelash, whose telephone is 0911261296 or email: sileyeshi21@gmail.com

2. Annex -2 Questionnaires

2.1. SURVEILLANCE SYSTEM EVALUATION REGION LEVEL QUESTIONNAIRE

IDENTIFIERS:

Assessor team Name/ID ______________________ Respondent's Name ________________

Responder's Phone number -------------------------- Responsibility ___________________________

Year of Experience ---------- Service years in this office ----------- Profession ----------------------

Date of assessment _________________ Interviewer Name & signature________________________

I. AVAILABILITY OF A NATIONAL SURVEILLANCE MANUAL

1. Is there a national manual for surveillance?

Yes No Unknown Not applicable

2. *If yes*, describe (last update, diseases included, case definitions, surveillance and control, integrated or different for each disease): ____

____

____

____

II. CASE DETECTION AND REGISTRATION

3. Do you have standard case definitions for the Country's priority diseases like AWD, AFP (polio), Malaria, SAM, Typhoid fever, Relapsing fever and measles?

1. Yes *2. No* *3. Unknown* *4. Not applicable*

4. *If other than yes to Q#- 3 above, describe their point of view* --

--------- --

5. Obs [One to 'n' priority diseases] Observed the standard case definition for (each priority disease). *1. Yes* *2. No* *3. Unknown* *4. Not applicable*

III. Data reporting: ________________

6. Presence of recommended reporting forms in the country at all times over the past 6 months?

1. Yes *2. No* *3. Unknown* *4. Not applicable*

7. If other than yes, describe --

8. Is the central level responsible for providing surveillance forms to the health facilities?

1. Yes *2. No* *3. Unknown* *4. Not applicable*

9. If other than yes, describe ---

10. *If yes*, have you lacked appropriate surveillance forms at any time during the last 6 months?

1. Yes *2. No* *3. Unknown* *4. Not applicable*

11. What are the reporting entities for the surveillance system?

1. Public health facilities *2.* NGO health facilities *3.* Military health facilities *4.* Private health facilities 5. Others__________________________

12. Number of facilities delivering health care in the region (Hosp, clinic, health center, temporary units) be it public or not---------------

13. Percent of district reports (either directly or through an intermediate level) received each reporting period at the central level during the past 3 months:

14. Number of reports in the last 3 months compared to expected number

Weekly: /12 times the number of districts

Immediately: /------ times the number of districts

15. On time (use national deadlines)

Number of weekly reports received on time: /12 times the number of districts

16. Was there any report of the immediately reportable diseases in the past 1 month?

Yes No

17. If yes, what amount of time it was/were required to detect the case?

1. <12hrs 2. <24hr 3. <72hr 4. < a week 5. < a month 6. Specify if other

18. If yes to Q16, with in what time is the report received after detection of the case/ diseases?

1. < one hour 2. (2-24) hrs. 3. (1- 2) days 4. (3- 7) days 5. After one week

19. Percent of districts that have means for reporting to next level by e-mail, telephone, fax or radio__________________________

20. Capacity to report to next level by e-mail, telephone, fax or radio: How do you report?

1. Mail Fax 2. Telephone 3.Radio 4. Electronics 5. Other(specify)----

IV. Data analysis

21. Does the region /city administrational/: Describe data by person check for all (case based, outbreaks, sentinel)? Observed description of data by age and Sex:

Yes No Unknown Not applicable

22. Describe their view if not yes for any of the three (case based, outbreaks, sentinel) ---

23. Describe data by place? Observed description of data by district (tables, maps)

Yes No Unknown Not applicable

24. Describe their view if not yes for any of the three (case based, outbreaks, sentinel) ---

25. Describe data by time? Observed description of data by time:

Yes, No Unknown Not applicable

26. Describe their view if not yes for any of the three (case based, outbreaks, sentinel) ---

27. Perform trend analysis? Observed line graph of cases by time

Yes, No, Unknown, Not applicable

28. List disease(s) for which line graph is observed ____

____

____

29. Has an action threshold defined for each priority disease? Do you have an action threshold defined for AWD, Measles, AFP (polio), ---------------, -------------, and Malaria? (Check for area specific diseases too).

Yes, No Unknown Not applicable

30. Please describe if answer to Q#29 is other than yes, -----------------------------------

31. Who is responsible for the analysis of the collected data? Why? ________________________

32. How often do you analyze the collected data?

Daily, Weekly, Every two weeks, Monthly, Quarterly, As needed

33. Do you have appropriate denominators? Observed presence of demographic data (E.g., population by district and hard to reach groups)

Yes, No Unknown Not Applicable

34. Please describe if answer to Q#29 is other than yes, --------------- -------------------

V. Outbreak Investigation

35. Percent of suspected outbreaks that were investigated in the past 6 months ----------

36. Number of outbreaks suspected in the past year: ________________________

37. List the diseases: ____

38. Of those, number investigated: ____ (Observed reports and take copies if possible)

39. Of the investigated outbreaks in the past 1 year, percent in which risk factors were looked for:

40. Number of outbreaks in which risk factors were used for: _______________

41. Percent in which findings were used for action [Observed report] ------------------------------

42. Of districts that investigated an outbreak, percent that looked for risk factors. Number of districts that looked for risk factors [observed in reports] ________________

43. Of districts that investigated an outbreak, percent that used the data for action (action include containing outbreak, improving surveillance, community actions)

44. Number of districts that used the data for action [observe in final report] _________________ ____

VI. EPIDEMIC PREPAREDNESS (RELEVANT FOR EPIDEMIC PRONE DISEASES)

45. Existence of a regional/city/administrational/plan for epidemic preparedness and response Obs Observed a written plan of epidemic preparedness and response

Yes, No Unknown Not applicable

46. If not yes, describe it --------------------------

47. Existence of emergency stocks of drugs, vaccines, and supplies at all times in past 1 year:

47.1. Has the administration had emergency stocks of drugs, vaccines, and supplies at all times in past 1 year?

Yes, No Unknown Not applicable

47.2. If not yes, describe it --------------------------

48. Experience of a shortage of drugs, vaccines or supplies during the most recent epidemic (or outbreak). Has the country experienced shortage of drugs, vaccines or supplies during the most recent epidemic (or outbreak)?

Yes, No Unknown Not applicable

49. If not yes, describe it --------------------------

50. Existence of a standard case management protocol for AWD, Malaria, AFP (polio), measles, ------------------, -------------- (Observed the existence of a written case management protocol for at least one priority disease)

51. If yes, list: ____

52. If no why? ---

53. Presence of a budget line for epidemic response Is there a budget line for epidemic response?

Yes, No Unknown Not applicable

54. If yes, enlighten the source -------------------------

55. If other than yes, why? ---

56. Existence of a regional/city/ administration epidemic management committee Observed minutes (or report) of meetings of epidemic management committee

Yes, No Unknown Not applicable

57. If yes, describe their composition? ------------------

58. If not yes, why? ------------------------------------

59. Existence of a regional/city/ administration rapid response team for epidemics?

Yes, No Unknown Not applicable

60. If yes, list compositions? ---------------------------

61. If not yes, describe--------------------------------

62. Do the RRT has regular meeting?

Yes, No Unknown Not applicable

63. If yes how often --------------------------

64. If not yes, define it -----------------------------

VII. RESPONSE TO EPIDEMICS:

Ability of the regional administrational level to respond within 48 hours of notification of most recently reported outbreak:

65. Observed that the central level responded within 48 hours of notification of most recently reported outbreak (from written reports with trend and intervention)

Yes, No Unknown Not applicable

66. If not yes, discuss it ----------------

67. Ability of the regional administrational epidemic management committee to evaluate its preparedness and response activities:

Has epidemic management committee evaluated its preparedness and response activities during the past year (Observed written report to confirm)?

Yes, No Unknown Not applicable

68. If yes, describe its best findings --------------------------------------

69. If not yes, why? ---

VIII. FEEDBACK:

Existence of a report or bulletin that is regularly produced to disseminate surveillance data:

70. How many feedback bulletin or reports has the regional/city administration level produced in the last year? ___________________

71. Observed the presence of a report or bulletin that is regularly produced to disseminate surveillance data.

Yes, No Unknown Not applicable

72. If yes, what was your mechanism ----------------------------

73. If not yes describe why & the way forwarded -----------------------------

IX. SUPERVISION:

Percent of supervisors that made the required number of supervisory visits in the past 6 months

74. Do you have a written annual supervision plan for lower-level sites (Observe)?

Yes, No Unknown Not applicable

75. If not yes, why? -----------------------------

76. Were your supervisions reasonably regular?

Yes, No Unknown Not applicable

77. How many supervisory visits have you made in the last 6 months? _____________________

78. The most usual reasons for not making all required supervisory visits. (Text)_______________

____

____ ________________

X. TRAINING;

Percent of health personnel trained in disease surveillance.

79. How many PHEM/surveillance/ personnel do you have in your subordinate structures? -------

80. What percent of your subordinate personnel have been trained in surveillance? ______

81. Have you been trained in disease surveillance?

Yes, No Unknown Not applicable

82. *If yes,* specify when, where, how long, by whom?______________________________________

____

____

Percent of health personnel that have received post-basic training in epidemic management

83. Have you received any post-basic training in epidemic management?

Yes, No Unknown Not applicable

84. If yes, specify when, where, how long, by whom? ____________________________________

____

85. Obtain and analyze the content of the surveillance and epidemic management training:

Strengths ____

Weaknesses ____

Opportunities ____

Threats ____

XI. Resources:

Percent of sites that have:

86. Data management tool

1. Computer, 2, Printer 3, Photocopier 4, Data manager 5, Statistical package

87. Communications media

1- Telephone 2- Fax 3, Radio call 4-Satellite phone 5- Computers that have modems 6. Other specify

88. Budget line ____

89. Logistics ____

XII. Surveillance:

Have a functional computerized surveillance network

90. Do you have a computerized surveillance network at this level?

Yes, No Unknown Not applicable

Budget for surveillance

91. Is there a budget line for surveillance in the region city/administrational Health Bureau budget?

Yes, No Unknown Not applicable

92. If yes, what is the proportion: _____________%

Opportunities for strengthening surveillance

93. How could surveillance be improved? ____

____

____

XIII. Surveillance Co-ordination

Existence of focal unit for surveillance at region level

94. Is there a focal unit for surveillance at the region level? [Observed organogram of the region to confirm]

Yes, No Unknown Not applicable

Opportunities for integration:

95. What opportunities are there for integration of surveillance activities and functions (core activities, training, supervision, guidelines, resources etc.)? --------------------------------------

____

____

____

96. *If no,* how can the surveillance system are improved? ___________________________________

____

____

97. Opportunities for integration _______________________________________ ________________

____ ___________________________

QUESTIONNAIRE FOR ATTRIBUTES AND LEVEL OF USEFULNESS:

1. Total population under surveillance______________
2. What is the incidence-- / Prevalence --- Deaths of -------in your area/Catchment

AWD ________cases _________Deaths ________

Malaria ________cases _________Deaths ________

AFP (polio) ________cases _________Deaths ________

Measles ________cases _________Deaths ________

SAM ________cases _________Deaths ________

________________ -

I. LEVEL OF USEFULNESS OF THE SURVEILLANCE SYSTEM FOR THESE SELECTED PRIORITY DISEASES.

1. Does the surveillance system help to detect outbreaks of these selected priority diseases early?

Yes No I don't know

2. If yes, how? --------------------------------
3. If no, why? --------------------------------

4. Does the surveillance system help to estimate the magnitude of morbidity and mortality related to these diseases, including identification of factors associated with these diseases?

   Yes No I don't know

5. If yes, how? --------------------------------
6. If no, why? --------------------------------
7. Does the system help to identify priority conditions & most at risk conditions?

   Yes No

8. If yes how? --------------
9. If no why? ----------------------
10. Does it help you to know the prevalence & incidence of the priority conditions in the area?

    Yes No

11. If yes, how?
12. If no, why?
13. Does it help as a background information to a regional action plan against and baseline data for a regional intervention Yes No
14. If yes, how? ______________________________
15. If no why? __________________________
16. Does the system stimulate any research related activities in the region?

    Yes No

17. If yes describe it ----------------------------
18. Does the system attract donors (NGO, Gov'I, local community) to improve itself?

    Yes No

19. Does the surveillance system help to permit assessment of the effect of prevention and control programs? Observed (confirmation): interventions and diseases trends analyzed ---Available /Not available Yes No I don't know

II. DESCRIBE EACH SYSTEM ATTRIBUTES:

A. SIMPLICITY:

1. Is the case definition of AWD, -------------, ----------, Malaria, AFP (polio), and Measles easy for case detection by all level health professionals? Yes No

2. If yes how? -------------------------
3. If no, why? ----------------------------
4. What are the organizations which need to receive reports of the surveillance data? _________ ____
5. Do you feel that additional data collected on a case are time consuming? Yes, No
6. How long it takes to fill the format?

   <5-minutes 10-15minuts >15 minutes
7. How long does it take to have laboratory confirmation of

   AWD _____, Measles ____, AFP (Polio) _____,

   Malaria ________, ________________, ______________
8. Is the principle of surveillance information utilization simple?

   Yes No
9. Is it difficult to run and coordinate the surveillance system in your area?

   Yes, No
10. If yes, how? ---------------------------------
11. If no, why? --------------------------------
12. Are instructions and guidelines for identifying cases, completing surveillance formats simple Yes, No
13. Clearly described the system procedures

    Yes No

B. FLEXIBILITY:

1. Can the current reporting formats be used for other newly occurring health event (disease) without much difficulty? Yes No
2. If yes, how? --------------------------
3. If no, why? ------------------------------------
4. Do you think that any change in the existing procedure of case detection, reporting, and formats will be difficult to implement? Yes No
5. If yes how? ---------------------------
6. If no, why? _________________
7. Which could possibly bring difficult problem in your surveillance system? Why?

   A, New health-related events, B. Changes in case definitions

C. Change in Technology E. Reporting sources

D. Variations in funding

8. Do the system allow more prevalence surveys per year and additional types of infections to be included at the local level

   Yes No

C. DATA QUALITY: (Completeness of the reporting forms/and validity of the recorded data)

1. Are the data collection formats for these priority diseases clear and easy to fill for all the data collectors/ reporting sites? Yes No
2. If yes how? --------------------------
3. If no, why? __________________
4. Are the reporting site / data collectors trained/ supervised regularly?

   Yes No
5. If no, why? ----------------------------
6. Observed: Review the last months report of these diseases
   A. Average number of unknown or blank responses to variables in each of the reported forms

      ____

      ____
   B. Reasons for not filling them ----------------------------------
   C. Percent of reports which are complete (that is with no blank or unknown responses) from the total reports ____

D. ACCEPTABILITY:

1. Do you think all the reporting agents accept and well engaged to the surveillance activities?

   Yes No
2. If yes, how? --------------------------
3. If yes, how many are active participants (of the expected to)? _______________
4. If no, what is the reason for their poor participation in the surveillance activity?

   A/ Lack of understanding of the relevance of the data to be collected

   B/ No feedback / or recognition given by the higher bodies for their contribution; i.e. no dissemination of the analysis data back to reporting facilities

   C/ Reporting formats are difficult to understand

D/ Report formats are time consuming

E/ Other: ____

5. Do the end-users are willing to accept and use data generated through the system?

   Yes No

6. If yes how? -------------------------------

7. If no, how? ----------------------------------

8. Do sites report meaningful and completeness reports?

   Yes No

9. Do surveillance personnel admit their contributions and inputs to the existing _ IDSR system were considered valuable

   Yes No

10. Do surveillance respondents demonstrated satisfaction with their involvement and agree for its PH importance?

    Yes No

11. If no why? ______________________

E. REPRESENTATIVENESS:

1. What is the health service coverage of the city/region administration? _________%
2. Do you think, the populations under surveillance have good health seeking behavior for these diseases? Yes No
3. If yes how? ------------------------
4. If no, why? ---------------------------------------
5. Who do you think which is well represented by the surveillance data?

   Urban Rural Both None

6. Why? ----------------------------

F. STABILITY:

1. Do you think your surveillance system has the ability to collect, manage, and provide data properly without failure in the future? Yes No

2. Was the new restructuring affect the procedures and activities of the surveillance of these diseases? Yes No
3. If yes, how? ______________________
4. If no, how?
5. Was there lack of resources that interrupt the surveillance system? Yes No
6. If yes, how? __________________
7. If no, why?
8. The number of unscheduled outages and down times for the system's computer in this year? --------
9. Do your data storage system safe & efficient? Yes No
10. If yes how? _____________________________
11. If no, why? _________________________
12. Do you have reasonable number of staff trained? Yes No
13. Do have acceptable items like (functional transport and/or communication equipment and stationery) avail ability at the lower level Yes No

G. TIMELINESS: -------------------

1. Are reporting units reporting on time? Yes No
2. If no why? ------------------------
3. Percent of units that report on time --------------------------
4. What time was/were required to notify the 3 most recent outbreaks in the area? -------------

Weekly regional reports received on time for 2022 report by WHO epidemic week to be field at regional health department level

WHO week	Expected					Reported				
	Zones	District	Hosp	HC	Clinic	Zones	District	Hosp	HC	Clinic

H. COMPLETENESS:

1. Are all units reporting including late report? Yes No
2. If no why? _______________________
3. Percent of units that send report of each week in 2014 EFY
4. Do reported cases reflect occurrence and distribution of all cases in the population under surveillance Yes No
5. If yes, how?
6. If no why?
7. Is the system applicable for any of the sites (urban or rural) or other socio demographic too and the system draws reports from the lowest (community 1-5 networks) level to the upper

   Yes No

I. PREDICTIVE VALUE POSITIVE

1. The proportion of epidemics identified by the surveillance system that are true epidemics---------------------------
2. what proportion of cases identified by your definition were confirmed as case by the standard confirmatory test for any outbreaks in your area

J. SENSITIVITY

1. Does your surveillance case-definitions sensitive enough to detect majority of cases in the community Yes No

2. If yes, how? ________________________

3. If no, how? _______________________

4. How many false positive cases were reported in your system? ---------------------

ZONAL/DISTRICT SURVEILLANCE SYSTEM EVALUATION CHECKLIST

I. IDENTIFIERS:

Assessor team Name/ID ____________________ Respondent's Name _______________

Responder's Phone number -------------------------- Responsibility ________________________

Year of Experience ---------- Service years in this office ----------- Profession ---------------------

Date of assessment ________________ Interviewer Name & signature______________________

II. AVAILABILITY OF A NATIONAL SURVEILLANCE MANUAL

1. Is there a national manual for surveillance?

   Yes, No Unknown Not applicable

2. *If yes*, describe (last update, diseases included, case definitions, surveillance and control, integrated or different for each disease): ______________________________________

   ____

   ____

   ____

III. CASE DETECTION AND REGISTRATION

1. Do you have standard case definitions for the Country's priority diseases like AWD, AFP (polio), Malaria, SAM, -------------, ---------------- and measles?

   Yes, No Unknown Not applicable

2. If other than yes to Q#- 3 above, describe their point of view --- ---------- ---

3. [One to 'n' priority diseases] Observed the standard case definition for (each priority disease).

   Yes, No Unknown Not applicable

IV. Data reporting: _______________

1. Presence of recommended reporting forms in the district at all times over the past 6 months?

   Yes, No Unknown Not applicable

2. If other than yes, describe --

3. Is the central level responsible for providing surveillance forms to the health facilities?

   Yes, No Unknown Not applicable

4. If other than yes, describe --

5. *If yes*, have you lacked appropriate surveillance forms at any time during the last 6 months?

   Yes, *No* *Unknown* *Not applicable*

6. What are the reporting entities for the surveillance system?

   Public HFs NGO HFs Military HFs Private HFs Others________

7. Number of facilities delivering health care in the district (Hosp, clinic, health center, temporary units) be it public or not---------------
8. Percent of HFs reports (either directly or through an intermediate level) received each reporting period at the central level during the past 3 months:
9. Number of reports in the last 3 months compared to expected number

   Weekly: /12 times the number of districts

   Immediately: /------ times the number of districts

10. Number of weekly reports received on time: /12 times the number of districts
11. Was there any report of the immediately reportable diseases in the past 1 month?

    Yes No

12. If yes, what amount of time it was/were required to detect the case?

    1. <24hr 2. <72hr 3. < a week 4. < a month 5. Specify if other

13. If yes to Q12, with in what time is the report received after detection of the case/ diseases?

    1. < one hour 2. (2-24) hrs. 3. (1- 2) days 4. (3- 7) days 5. After one week

14. Percent of districts that have means for reporting to next level by e-mail, telephone, fax or radio_________________________
15. Capacity to report to next level by e-mail, telephone, fax or radio: How do you report?

    1. Mail Fax 2. Telephone 3.Radio 4. Electronics 5. Other(specify)----

V. Data analysis

1. Does the district: Describe data by person check for all (case based, outbreaks, sentinel)? Observed description of data by age and Sex:

   Yes, No Unknown Not applicable

2. Describe their view if not yes for any of the three (case based, outbreaks, sentinel) -------------
   ----
3. Describe data by place? Observed description of data by district (tables, maps)

   Yes, No Unknown Not applicable

4. Describe their view if not yes for any of the three (case based, outbreaks, sentinel) ------------- --

5. Describe data by time? Observed description of data by time:

   Yes, No Unknown Not applicable

6. Describe their view if not yes for any of the three (case based, outbreaks, sentinel) ------------- --

7. Perform trend analysis? Observed line graph of cases by time

   Yes, No Unknown Not applicable

8. List disease(s) for which line graph is observed ____ ____ __________________________________

9. Has an action threshold defined for each priority disease? Do you have an action threshold defined for AWD, Measles, AFP (polio), ----------------, --------------, and Malaria? (Check for area specific diseases too).

   Yes, No Unknown Not applicable

10. Please describe if answer to Q#29 is other than yes, -----------------------------------

11. Who is responsible for the analysis of the collected data? Why? _______________________

12. How often do you analyze the collected data?

    Daily, Weekly, Every two weeks, Monthly, Quarterly, As needed

13. Do you have appropriate denominators? Observed presence of demographic data (E.g., population by district and hard to reach groups)

    Yes, No Unknown Not Applicable

14. Please describe if answer to Q#13 is other than yes, ---------------- --------------------

VI. OUTBREAK INVESTIGATION

1. Percent of suspected outbreaks that were investigated in the past 6 months ----------
2. Number of outbreaks suspected in the past year: _______________________
3. List the diseases: ____
4. Of those, number investigated: ____ (Observed reports and take copies if possible)
5. Of the investigated outbreaks in the past 1 year, percent in which risk factors were looked for:
6. Number of outbreaks in which risk factors were used for: ______________

7. Percent in which findings were used for action [Observed report] ------------------------------
8. Of districts that investigated an outbreak, percent that looked for risk factors. Number of districts that looked for risk factors [observed in reports] ________________
9. Of districts that investigated an outbreak, percent that used the data for action (action include containing outbreak, improving surveillance, community actions)
10. Number of districts that used the data for action [observe in final report] ________________ ____

VII. EPIDEMIC PREPAREDNESS (RELEVANT FOR EPIDEMIC PRONE DISEASES)

1. Existence of a district/city/administrational/plan for epidemic preparedness and response
   Obs Observed a written plan of epidemic preparedness and response
   Yes, No Unknown Not applicable
2. If not yes, describe it --------------------------
3. Existence of emergency stocks of drugs, vaccines, and supplies at all times in past 1 year:
4. Has the administration had emergency stocks of drugs, vaccines, and supplies at all times in past 1 year? Yes, No Unknown Not applicable
5. If not yes, describe it --------------------------
6. Experience of a shortage of drugs, vaccines or supplies during the most recent epidemic (or outbreak). Has the country experienced shortage of drugs, vaccines or supplies during the most recent epidemic (or outbreak)?
   Yes, No Unknown Not applicable
7. If not yes, describe it --------------------------
8. Existence of a standard case management protocol for AWD, Malaria, AFP (polio), measles, ------------------, -------------- (Observed the existence of a written case management protocol for at least one priority disease)
9. If yes, list: ____
10. If no why? --
11. Presence of a budget line for epidemic response Is there a budget line for epidemic response?
    Yes, No Unknown Not applicable
12. If yes, enlighten the source -------------------------
13. If other than yes, why? --

14. Existence of a district epidemic management committee Observed minutes (or report) of meetings of epidemic management committee

    Yes, No Unknown Not applicable

15. If yes, describe their composition? ------------------
16. If not yes, why? ------------------------------------
17. Existence of a district rapid response team for epidemics?

    Yes, No Unknown Not applicable

18. If yes, list compositions? ---------------------------
19. If not yes, describe--------------------------------
20. Do the RRT has regular meeting?

    Yes, No Unknown Not applicable

21. If yes how often --------------------------
22. If not yes, define it -----------------------------

VIII. RESPONSE TO EPIDEMICS:

Ability of the district to respond within 48 hours of notification of most recently reported outbreak:

1. Observed that the district responded within 48 hours of notification of most recently reported outbreak (from written reports with trend and intervention)

    Yes, No Unknown Not applicable

2. If not yes, discuss it ----------------
3. Has epidemic management committee evaluated its preparedness and response activities during the past year (Observed written report to confirm)?

    Yes, No Unknown Not applicable

4. If yes, describe its best findings ---------------------------------------
5. If not yes, why? --

IX. FEEDBACK:

Existence of a report or bulletin that is regularly produced to disseminate surveillance data:

1. How many feedback bulletin or reports has the district produced in the last year? ___________________
2. Observed the presence of a report or bulletin that is regularly produced to disseminate surveillance data. Yes, No Unknown Not applicable
3. If yes, what was your mechanism ---------------------------

4. If not yes describe why & the way forwarded ----------------------------

X. SUPERVISION:

1. Percent of supervisors that made the required number of supervisory visits in the past 6 months
2. Do you have a written annual supervision plan for lower-level sites (Observe)?

   Yes, No Unknown Not applicable

3. If not yes, why? ----------------------------
4. Were your supervisions reasonably regular?

   Yes, No Unknown Not applicable

5. How many supervisory visits have you made in the last 6 months? ____________________
6. The most usual reasons for not making all required supervisory visits. (Text)______________

   ____

   ____ ______________

XI. TRAINING:

1. Percent of health personnel trained in disease surveillance.
2. How many PHEM/surveillance/ personnel do you have in your subordinate structures? ---
3. What percent of your subordinate personnel have been trained in surveillance? ______
4. Have you been trained in disease surveillance?

   Yes, No Unknown Not applicable

5. *If yes*, specify when, where, how long, by whom?_______________________________________

   ____

   ____

6. Percent of health personnel that have received post-basic training in epidemic management
7. Have you received any post-basic training in epidemic management?

   Yes, No Unknown Not applicable

8. If yes, specify when, where, how long, by whom? __________________________________

   ____

9. Obtain and analyze the content of the surveillance and epidemic management training:

   Strengths ____

   Weaknesses ______________________________________

   Opportunities ______________________________________

   Threats ____

XII. Resources:

Percent of sites that have:

1. Data management tool

   Computer, Printer Photocopier Data-Manager Statistical-package

2. Communications media

   Telephone Fax Radio call Satellite phone Other specify

3. Budget line ____
4. Logistics ____
5. Do you have a computerized surveillance network at this level?

   Yes, No Unknown Not applicable

6. Is there a budget line for surveillance in the district city/administrational Health Bureau budget? Yes, No Unknown Not applicable
7. If yes, what is the proportion: _____________%

XIII. Opportunities for strengthening surveillance

1. How could surveillance be improved? ____
   ____
   ____

XIV. Surveillance Co-ordination

Existence of focal unit for surveillance at district level

1. Is there a focal unit for surveillance at the district level? [Observed organogram of the district to confirm]

   Yes, No Unknown Not applicable

Opportunities for integration:

2. What opportunities are there for integration of surveillance activities and functions (core activities, training, supervision, guidelines, resources etc.)? -------------------------------------
   ____
   ____
   ____

3. *If no*, how can the surveillance system are improved? ________________________________
____
____

4. Opportunities for integration ____________________________________ _______________
____   _________________________

QUESTIONNAIRE FOR ATTRIBUTES AND LEVEL OF USEFULNESS:

1- Total population under surveillance_____________

2- What is the incidence-- / Prevalence --- Deaths of -------in your area/Catchment

AWD ________cases _________Deaths ________

Malaria ________cases _________Deaths ________

AFP (polio) ________cases _________Deaths ________

Measles ________cases _________Deaths ________

SAM ________cases _________Deaths ________

_______________ -

I. LEVEL OF USEFULNESS OF THE SURVEILLANCE SYSTEM FOR THESE SELECTED PRIORITY DISEASES.

1. Does the surveillance system help to detect outbreaks of these selected priority diseases early?

   Yes No I don't know

2. If yes, how? --------------------------------
3. If no, why? --------------------------------
4. Does the surveillance system help to estimate the magnitude of morbidity and mortality related to these diseases, including identification of factors associated with these diseases?

   Yes No I don't know

5. If yes, how? --------------------------------
6. If no, why? --------------------------------
7. Does the system help to identify priority conditions & most at risk conditions?

   Yes No

8. If yes how? -------------

9. If no why? ---------------------
10. Does it help you to know the prevalence & incidence of the priority conditions in the area?

    Yes No

11. If yes, how? ___________________
12. If no, why? ______________________
13. Does it help as a background information to a district action plan against and baseline data for a district intervention Yes No
14. If yes, how? ______________________________
15. If no why? __________________________
16. Does the system stimulate any research related activities in the district?

    Yes No

17. If yes describe it ----------------------------
18. Does the system attract donors (NGO, Gov'I, local community) to improve itself?

    Yes No

19. Does the surveillance system help to permit assessment of the effect of prevention and control programs? Observed (confirmation): interventions and diseases trends analyzed ---Available /Not available Yes No I don't know

DESCRIBE EACH SYSTEM ATTRIBUTES:

I. SIMPLICITY:

1- Is the case definition of AWD, -------------, ----------, Malaria, AFP (polio), and Measles easy for case detection by all level health professionals? Yes No

2- If yes how? -------------------------

3- If no, why? ----------------------------

4- What are the organizations which need to receive reports of the surveillance data? _________ ____

5- Do you feel that additional data collected on a case are time consuming? Yes, No

6- How long it takes to fill the format? <5-minutes 10-15minutes, >15 minutes

7- How long does it take to have laboratory confirmation of

   AWD _____, Measles ____, AFP (Polio) _____,

Malaria ________, ________________, _____________

8- Is the principle of surveillance information utilization simple?

Yes No

9- Is it difficult to run and coordinate the surveillance system in your area?

Yes, No

10- If yes, how? ---------------------------------

11- If no, why? --------------------------------

12- Are instructions and guidelines for identifying cases, completing surveillance formats simple

Yes, No

13- Clearly described the system procedures

Yes No

II. FLEXIBILITY:

14- Can the current reporting formats be used for other newly occurring health event (disease) without much difficulty? Yes No

15- If yes, how? -------------------------

16- If no, why? ----------------------------------

17- Do you think that any change in the existing procedure of case detection, reporting, and formats will be difficult to implement? Yes No

18- If yes how? --------------------------

19- If no, why? _________________

20- Which could possibly bring difficult problem in your surveillance system? Why?

A, New health-related events, D. Variations in funding

B. Changes in case definitions E. Reporting sources

C. Change in Technology

21- Do the system allow more prevalence surveys per year and additional types of infections to be included at the local level

Yes No

III. DATA QUALITY: (Completeness of the reporting forms/and validity of the recorded data)

22- Are the data collection formats for these priority diseases clear and easy to fill for all the data collectors/ reporting sites? Yes No

23- If yes how? --------------------------

24- If no, why? _________________

25- Are the reporting site / data collectors trained/ supervised regularly?

Yes No

26- If no, why? ----------------------------

27- Average number of unknown or blank responses to variables in each of the reported forms (Observed: Review the last months report of these diseases) __________________________

28- Reasons for not filling them ----------------------------------

29- Percent of reports which are complete (that is with no blank or unknown responses) from the total reports ____

IV. ACCEPTABILITY:

30- Do you think all the reporting agents accept and well engaged to the surveillance activities?

Yes No

31- If yes, how? --------------------------

32- If yes, how many are active participants (of the expected to)? _____________

33- If no, what is the reason for their poor participation in the surveillance activity?

A/ Lack of understanding of the relevance of the data to be collected

B/ No feedback / or recognition given by the higher bodies for their contribution; i.e. no dissemination of the analysis data back to reporting facilities

C/ Reporting formats are difficult to understand

D/ Report formats are time consuming

E/ Other: ____

34- Do the end-users are willing to accept and use data generated through the system?

Yes No

35- If yes how? -------------------------------

36- If no, how? ----------------------------------

37- Do sites report meaningful and completeness reports?

Yes No

38- Do surveillance personnel admit their contributions and inputs to the existing _ IDSR system were considered valuable? Yes No

39- Do surveillance respondents demonstrated satisfaction with their involvement and agree for its PH importance? Yes No

40- If no why? ______________________

V. REPRESENTATIVENESS:

41- What is the health service coverage of the city/district administration? _________%

42- Do you think, the populations under surveillance have good health seeking behavior for these diseases? Yes No

43- If yes how? ------------------------

44- If no, why? --------------------------------------

45- Who do you think which is well represented by the surveillance data?

Urban Rural Both None

46- Why? ----------------------------

VI. STABILITY:

47- Do you think your surveillance system has the ability to collect, manage, and provide data properly without failure in the future? Yes No

48- Was the new restructuring affect the procedures and activities of the surveillance of these diseases? Yes No

49- If yes, how? ____________________

50- If no, how?

51- Was there lack of resources that interrupt the surveillance system? Yes No

52- If yes, how? ________________

53- If no, why?

54- The number of unscheduled outages and down times for the system's computer in this year? --------

55- Do your data storage system safe & efficient? Yes No

56- If yes how? __________________________

57- If no, why? _______________________

58- Do you have reasonable number of staff trained?   Yes   No

59- Do have acceptable items like (functional transport and/or communication equipment and stationery) avail ability at the lower level   Yes   No

VII. TIMELINESS: -------------------

60- Are reporting units reporting on time?   Yes   No

61- If no why? ------------------------

62- Percent of units that report on time --------------------------

63- What time was/were required to notify the 3 most recent outbreaks in the area? --------------

Weekly districtal reports received on time for 2022 report by WHO epidemic week to be field at districtal health department level

WHO week	Expected					Reported				
	District	Hosp	HC	Clinic	HP	District	Hosp	HC	Clinic	HP

VIII. COMPLETENESS:

64- Are all the units report including late report? Yes   No

65- If no why? ________________________

66- Percent of units that send report of each week in 2014 EFY ---------------

67- Do reported cases reflect occurrence and distribution of all cases in the population under surveillance   Yes   No

68- If yes, how? ___________________________

69- If no why? _________________________

70- Is the system applicable for any of the sites (urban or rural) or other socio demographic too and the system draws reports from the lowest (community 1-5 networks) level to the upper
Yes   No

IX. PREDICTIVE VALUE POSITIVE

71- The proportion of epidemics identified by the surveillance system that are true epidemics-----

72- what proportion of cases identified by your definition were confirmed as case by the standard confirmatory test for any outbreaks in your area?

IX. SENSITIVITY

73- Does your surveillance case-definitions sensitive enough to detect majority of cases in the community Yes No

74- If yes, how? ________________________

75- If no, how? _______________________

76- How many false positive cases were reported in your system? ---------------------

SURVEILLANCE SYSTEM EVALUATION HOSPITAL /HEALTH CENTER QUESTIONNAIRE

I. IDENTIFIERS

Assessor team ------------------------ Type of health facility-----------------

Date -------------------   District ------------------------------

Interviewer -----------------------------   District/province ------------------------

Respondent --------------------   Country -----------------

Name of health facility --------------------------

II. SURVEILLANCE SYSTEM

Percent of health facilities with national surveillance manual

1. Is there a national manual for surveillance at this site?

[Obs] Observed national surveillance manual:

Yes   No   Unknown   Not applicable

2. *If yes*, describe (last update, diseases included, case definitions, surveillance and control, integrated or different for each disease): ____

____

____

____

3. If no why? ----------------------

III. CASE DETECTION AND REGISTRATION__

Percent of health facilities that have a clinical register

3. Is there a clinical register? Observed the existence of a clinical register

Yes   No   Unknown   Not-applicable

4. Percent of health facilities that correctly register cases

[obs] Observed the correct filling of the clinical register during the previous 30 days

Yes   No   Unknown   Not applicable

5. Percent of health facilities that have standardized case definitions for the country's priority diseases. Do you have a standard case definition for: (each priority disease) AWD, AFP (polio), ------------, ----------------, Measles, Malaria?

Yes No Unknown Not applicable

6. If not yes, describe -------------------------------
7. Observed the standard case definition for: (each priority disease)

Yes No Unknown Not applicable

8. Percent of health facilities that use standardized case definitions for the country's priority diseases.

   Observed the respondent correctly diagnosing one of the country's priority diseases using a standard case definition (Select one of the priority diseases in the facility's clinical register and ask how they diagnosed it — interviewer should have the standard case definition from MOH)

   Yes No Unknown Not applicable

   9. *Percentage of case definitions understood by professionals working at case identification site?* ------------------------

IV. CASE CONFIRMATION ___________________

10. *P*ercent of health facilities that have the capacity to collect specimens (sputum stool, blood/serum and CSF). At this facility are you able to collect

Sputum	Y	N	U	N/A
Stool	Y	N	U	N/A
Blood	Y	N	U	N/A
CSF	Y	N	U	N/A

11. If not yes to any of the above please describe it & what alternative they have? --
12. Observed the presence of materials required to collect

Stool	Y	N	U	N/A
Blood / serum	Y	N	U	N/A
CSF	Y	N	U	N/A
Sputum	Y	N	U	N/A

13. If not yes to any of the above please describe it & what alternative they have? --
14. Percent of health facilities that have the capacity to handle specimens until shipment

Do you have the capacity to handle sputum, stool, blood/serum and CSF until shipment at this facility? Yes No Unknown Not applicable

15. Observed presence of functional cold chain at health facility

    Yes No Unknown Not applicable

16. Percent of health facilities that have the capacity to ship specimens to higher level laboratories. Observed presence of transport media for stool at health facility

    Yes No Unknown Not applicable

17. [Obs]Observed presence of packing materials for shipment of specimens at health facility Yes No Unknown Not applicable

V. DATA REPORTING ______________________

18. Percent of sites that have appropriate surveillance forms for that site at all times over the past 6 months. Have you lacked appropriate surveillance forms at any time during the last 6 months?

    Yes, No Unknown Not applicable

19. If other than yes, describe --
20. Is the central level responsible for providing surveillance forms to the health facilities?

    Yes No Unknown Not applicable

21. If other than yes, describe --
22. *If yes*, have you lacked appropriate surveillance forms at any time during the last 6 months?

    Yes No Unknown Not applicable

23. What are the reporting entities for the surveillance system?

    Public HFs NGO HFs Military HFs Private HFs Others________

24. Number of facilities delivering health care in the district (Hosp, clinic, health center, temporary units) be it public or not----------------
25. Was there any report of the immediately reportable diseases in the past 1 month?

    Yes No

26. If yes, what amount of time it was/were required to detect the case?

    1. <24hr 2. <72hr 3. < a week 4. < a month 5. Specify if other

25. If yes to Q12, with in what time is the report received after detection of the case/ diseases?

    1. < one hour 2. (2-24) hrs. 3. (1- 2) days 4. (3- 7) days 5. After one week

26. Percent of HFs reports (either directly or through an intermediate level) received each reporting period at the central level during the past 3 months:
27. Percent of sites that reported accurately cases from the registry into the summary report to go to higher level

Observed that the last monthly report agreed with the register for 4 diseases at least (1 for each targeted group [eradication; elimination; epidemic prone; major public health importance]) observed for

Measles	Y	N	U	N/A
Malaria	Y	N	U	N/A
AFP (polio)	Y	N	U	N/A
AWD	Y	N	U	N/A

28. Percent of sites that reported each reporting period to the next higher level during the past 3 months. Number of reports in the last 3 months compared to expected number

Obs Weekly: /12 times the number of sites

Obs immediately: /-- times the number of sites

On time (use national deadlines)

29. Obs Number of weekly reports submitted on time:_____ /12 times the number of sites
30. Obs Number of immediately reports submitted on time: ___/-- times the number of sites
31. Percent of HF that have means for reporting to next level by e-mail, telephone, fax or radio How do you report?

Mail Fax Telephone Radio Electronic Other

32. Strengthening reporting.

How can reporting be improved? ______________________ __________________

____

____

VI. DATA ANALYSIS_____________________

33. Percent of sites that: Describe data by person (Case based, outbreaks, sentinel)----
34. Observed description of data by age and sex

Yes No Unknown Not applicable

35. Describe their view if not yes for any of the three (case based, outbreaks, sentinel) -------------
----

36. Describe data by place

Observed description of data by place (locality, village, work site etc)

Yes No Unknown Not applicable

37. Describe their view if not yes for any of the three (case based, outbreaks, sentinel) --

38. Describe data by time Observed description of data by time

Yes No Unknown Not applicable

39. Perform trend analysis, Observed line graph of cases by time

Yes No Unknown Not applicable

40. List disease(s) for which line graph is observed ___________________________________ ____ ____________________________________

41. Have an action threshold for each priority disease? Do you have an action threshold for any of the Country priority diseases?

Yes No Unknown Not applicable

42. If yes, what is it (Ask for 2 priority diseases)? _______cases ____ % increase _____rate

43. Please describe if answer to Q#41 is other than yes, ----------------------------------

44. Who is responsible for data analysis? ___________________________

45. How often do you analyze the collected data?

Daily Weekly Every2 weeks Monthly Quarterly As needed

46. Have appropriate denominators Observed presence of demographic data at site (E.g. population <5yr., population by village, total population)

` Yes No Unknown Not applicable

47. Please describe if answer to Q#13 is other than yes, ---------------- -------------------

VII. OUTBREAK INVESTIGATION

48. Have you participated in outbreak investigation?

Yes No

49. Where when & on what? --------------------------------

50. Was/were there suspected outbreak detected in your catchment?

Yes No

51. Number of outbreaks suspected in the past year: _______________________
52. List the diseases: ____
53. Of the investigated outbreaks in the past 1 year, percent in which risk factors were looked for:
54. Number of outbreaks in which risk factors were used for action: ______________
55. Percent in which findings were used for action [Observed report] ------------------------------

VIII. EPIDEMIC PREPAREDNESS__________

56. Percent of health facilities that have a standard case management protocol for epidemic prone diseases, observed the existence of a written case management protocol for 1 epidemic prone disease.

Yes No Unknown Not applicable

57. Existence of a health facility plan for epidemic preparedness and response

Obs Observed a written plan of epidemic preparedness and response

Yes, No Unknown Not applicable

58. If not yes, describe it --------------------------
59. Existence of emergency stocks of drugs, vaccines, and supplies at all times in past 1 year:
60. Has the facility had emergency stocks of drugs, vaccines, and supplies at all times in past 1 year? Yes, No Unknown Not applicable
61. If not yes, describe it --------------------------
62. Experience of a shortage of drugs, vaccines or supplies during the most recent epidemic (or outbreak). Has the facility experienced shortage of drugs, vaccines or supplies during the most recent epidemic (or outbreak)?

Yes, No Unknown Not applicable

63. If not yes, describe it --------------------------
64. Presence of a budget line for epidemic response Is there a budget line for epidemic response?

Yes, No Unknown Not applicable

65. If yes, enlighten the source -------------------------
66. If other than yes, why? --

67. Existence of a facility epidemic management committee Observed minutes (or report) of meetings of epidemic management committee

Yes, No Unknown Not applicable

68. If yes, describe their composition? ------------------
69. If not yes, why? ------------------------------------
70. Existence of a district rapid response team for epidemics (RRT)?

Yes, No Unknown Not applicable

71. If yes, list compositions? ----------------------------
72. If not yes, describe---------------------------------
73. Do the RRT has regular meeting?

Yes, No Unknown Not applicable

74. If yes how often ---------------------------
75. If not yes, define it ------------------------------

IX. EPIDEMIC RESPONSE_______________

Percent of sites that implemented prevention and control measures based on local data for at least one epidemic prone disease

76. Has the health facility implemented prevention and control measures based on local data for at least one epidemic prone disease?

Yes No Unknown Not applicable

77. If not yes, discuss it -----------------
78. Has epidemic management committee evaluated its preparedness and response activities during the past year (Observed written report to confirm)?

Yes, No Unknown Not applicable

79. If yes, describe its best findings ---------------------------------------
80. If not yes, why? --
81. Percent of sites that achieved acceptable case fatality rates (e.g. 10% for Meningococcal CSM 1% for Cholera) during the most recent outbreak

Observed that the health facility achieved an acceptable case fatality rate for most recent outbreak

Yes No Unknown Not applicable

82.FEEDBACK ____________________

Percent of sites that have received a report or bulletin from a higher level during the past year on the data they have provided

83. How many feedback bulletin or reports has the health facility received in the last year? _________.

84. Observed at least 1 report or bulletin at the health facility from a higher level during the past year on the data they have provided.

    Yes No Unknown Not applicable

85. How many feedback reports has the facility produced in the last year? ___________________

86. Observed the presence of a report or bulletin that is regularly produced to disseminate surveillance data. Yes, No Unknown Not applicable

87. If yes, what was your mechanism ----------------------------

88. If not yes describe why & the way forwarded -----------------------------

89. Percent of health facilities that conducted at least semi-annual meetings with community members to discuss results of surveillance or investigation data __________

90. How many meetings has this health facility conducted with the community members in the past six months? ________________

91. Observed the minutes or report of at least 1 meeting between the health facility team and the community members within the six months. Yes No Unknown Not applicable

X. SUPERVISION: ____________________

Percent of individuals supervised in the past 6 months

92. How many times have you been supervised in the last 6 months?_________

93. Obs observed supervision report or any evidence of supervision in last 6 months.

    Yes No Unknown Not applicable

Of those supervised in the previous 6 months, percent of individuals for which the supervisor from the next higher level reviewed surveillance practices appropriate to their level.

94. [Obs] observed supervision report or any evidence for appropriate review of surveillance practices.

    Yes No Unknown Not applicable

95. Do you have supervision plan? Yes No Not Applicable

96. If not yes, why? ----------------------------

97. How many supervisory visits have you made in the last 6 months? ____________________

98. Were your supervisions reasonably regular?

Yes, No Unknown Not applicable

99. The most usual reasons for not making all required supervisory visits. (Text)______________

____

____ ________________

XI. TRAINING __________________

Percent of health personnel trained in disease surveillance and epidemic management

100. Have you been trained in disease surveillance and epidemic management?

Yes No Unknown Not applicable

101. If yes, specify when, where, how long, by whom?_____________________

____

____

XII. RESOURCES_______________

102. Percent of sites that have: Logistics

A. Electricity B. Bicycles C. Motor cycles D. Vehicles

103. Data management:

A. Stationery B. Calculator C. Computer

D. Software E. Printer F. Statistical package

104. Communications:

A. Telephone service C. Fax

B. Radio call D. Computers that have modems

105. Information education and communication materials:

A/ Posters B/ Megaphone C/ Flipcharts or Image box

D/ VCR and TV set E/ Generator F/ Screen

G/ Projector (Movie) H/ Other: (specify) ________________

106. Hygiene and sanitation materials:

A/ Spray pump B/ Disinfectant

107. Protection-materials (list) ____

____

___ ____

108. Is there a budget line for surveillance in the Health facility budget list? Yes, No Unknown Not applicable

109. If yes, what is the proportion: ____________%

SATISFACTION WITH SURVEILLANCE SYSTEM____

110. Are you satisfied with the surveillance system?

Yes No Unknown Not applicable

111. *If no*, how can the surveillance system be improved? _____________

____

____

112. Opportunities for integration

What opportunities are there for integration of surveillance activities and functions (core activities, training, supervision, guidelines, resources etc.

____

____

113. *If no*, how can the surveillance system are improved? ______________________________

____

____

114. Opportunities for integration ________________________________ ______________ ____

____

PART TWO

I. QUESTIONNAIRE FOR ATTRIBUTES AND LEVEL OF USEFULNESS:

1. Total population under surveillance_____________
2. What is the incidence-- / Prevalence --- Deaths of -------in your area/Catchment

________ ________cases _________Deaths ________

________ ________cases _________Deaths ________

_______ ________cases _________Deaths ________

II. LEVEL OF USEFULNESS OF THE SURVEILLANCE SYSTEM FOR THESE SELECTED PRIORITY DISEASES

3. Does the surveillance system help to detect outbreaks of these selected priority diseases early?

Yes No

4. If yes, how? --------------------------------

5. If no, why? --------------------------------

6. Does the surveillance system help to estimate the magnitude of morbidity and mortality related to these diseases, including identification of factors associated with these diseases?

Yes No

7. If yes, how? --------------------------------

8. If no, why? --------------------------------

9. Does the system help to identify priority conditions & most at risk conditions?

Yes No

10. If yes how? -------------

11. If no why? ---------------------

12. Does the surveillance system help to permit assessment of the effect of prevention and control programs? Yes No

Observed (confirmation): interventions and diseases trends analyzed ---Available / Not available

13. Does it help as a background information to a district action plan against and baseline data for a district intervention Yes No

14. If yes, how? ______________________________

15. If no why? __________________________

16. Does the system stimulate any research related activities in the district?

Yes No

17. If yes describe it ----------------------------

18. Does the system attract donors (NGO, Gov'I, local community) to improve itself?

Yes No

DESCRIBE EACH SYSTEM ATTRIBUTES:

A. SIMPLICITY:

19. Is the case definition of __________, _________________, ___________, Malaria, AFP (polio), and Measles easy for case detection by all level health professionals?

Yes No

20. If yes how? -------------------------

21. If no, why? ----------------------------

22. What are the organizations which need to receive reports of the surveillance data? ________________________________

23. Do you feel that additional data collected on a case are time consuming? Yes No

24. How long it takes to fill the format?

a, <5 minute b-10-15minuts c- >15 minutes

25. How long does it take to have laboratory confirmation of

AWD _____, Measles ____, AFP (Polio) _____,

Malaria ________, ------------- ___________, ------- _____,

26. Is the principle of surveillance information utilization simple?

Yes No

27. Is it difficult to run and coordinate the surveillance system in your area?

Yes, No

28. If yes, how? ---------------------------------

29. If no, why? --------------------------------

30. Are instructions and guidelines for identifying cases, completing surveillance formats simple Yes, No

B. FLEXIBILITY:

31. Can the current reporting formats be used for other newly occurring health event (disease) without much difficulty? Yes No

32. If yes, how? -------------------------

33. If no, why? -----------------------------------

34. Do you think that any change in the existing procedure of case detection, reporting, and formats will be difficult to implement? Yes No

35. If yes how? --------------------------

36. If no, why? _________________

37. Which do you think could possibly bring difficult problem in your surveillance system? Why?

New health-related events, Variations in funding

Changes in case definitions Reporting sources

Change in Technology

38. Does the system allow more prevalence surveys per year and additional types of infections to be included at the local level? Yes No

C. DATA QUALITY: _________________________

(Completeness of the reporting forms/and validity of the recorded data)

39. Are the data collection formats for these priority diseases clear and easy to fill for all the data collectors/ reporting sites? Yes No

40. If yes how? --------------------------

41. If no, why? _________________

42. Are the reporting site / data collectors trained/ supervised regularly? Yes No

43. If no, why? ______________________

44. Observed: Review the last months report of these diseases

Average number of unknown or blank responses to variables in each of the reported forms

____

____

Percent of reports which are complete (that is with no blank or unknown responses) from the total reports ____ __________________

45. Reasons for not filling them ____________________

D. ACCEPTABILITY:

46. Do you think all the reporting agents accept and well engaged to the surveillance activities?

Yes No

47. If yes, how many are active participants (of the expected to)? _____________

48. If no, what is the reason for their poor participation in the surveillance activity?

A/ Lack of understanding of the relevance of the data to be collected

B/ No feedback / or recognition given by the higher bodies for their contribution; i.e. no dissemination of the analysis data back to reporting facilities

C/ Reporting formats are difficult to understand

D/ Report formats are time consuming

E/ Other: ____

49. Do the end-users are willing to accept and use data generated through the system?

Yes No

50. If yes how? ------------------------------

51. If no, how? ---------------------------------

52. Do sites report meaningful and completeness reports?

Yes No

53. Do surveillance personnel admit their contributions and inputs to the existing _ IDSR system were considered valuable? Yes No

54. Do surveillance respondents demonstrated satisfaction with their involvement and agree for its PH importance? Yes No

55. If no why? ____________________

E. REPRESENTATIVENESS:

56. What is the health service coverage of the district/ zone/city administration? _________%

57. Do you think, the populations under surveillance have good health seeking behavior for these diseases? Yes No

58. If yes how? -----------------------

59. If no, why? -------------------------------------

60. Who do you think which is well represented by the surveillance data?

Urban Rural Both None

61. Why? ________________________

F. STABILITY:

62. Do you think your catchment surveillance system has the ability to collect, manage, and provide data properly without failure in the future? Yes No

63. Was the new restructuring affect the procedures and activities of the surveillance of these diseases? Yes No

64. If yes, how? _____________________

65. If no, how? ________________________

66. Was there an experience of lack of resources that interrupt the surveillance system?

Yes No

67. If yes, how? ________________

68. Do your data storage system safe & efficient? Yes No

69. If yes how? ___________________________

70. If no, why? ________________________

71. Do you have reasonable number of staff trained? Yes No

72. Do you have acceptable items like (functional transport and/or communication equipment and stationery) avail ability at the lower level Yes No

G. TIMELINESS: ------------------

73. Are the clinics & Health posts report on time? *Yes* *No*

74. *If no, why?* ___________________________

75. Percent of units report on time ----

76. What time was required to detect cases of any of your previous outbreaks ----------

77. What time was required to notify the detected event to the immediate next level -----

78. What time was required to start response to the detected event ___________

Weekly HFs reports received on time for 2022 report by WHO epidemic week to be field at district health department

WHO week	Expected					Reported				
	HC OPD	HC IPD	HC Other	Clinic	HP	HC OPD	HC IPD	HC Other	Clinic	HP

H. COMPLETENESS:

79. Are all the units report including late report? Yes No

80. If no, why? _______________

81. Percent of facilities that send report of each week in 2022

82. Do reported cases reflect occurrence and distribution of all cases in the population under surveillance Yes No

83. If yes, how? __________________________

84. If no why? _________________________

85. Is the system applicable for any of the sites (urban or rural) or other socio demographic too and the system draws reports from the lowest (community 1-5 networks) level to the upper

Yes No

I. PREDICTIVE VALUE POSITIVE

86. The proportion of epidemics identified by the surveillance system that are true epidemics--

87. what proportion of cases identified by your definition were confirmed as case by the standard confirmatory test for any outbreaks in your area?

J. SENSITIVITY

88. Does your surveillance case-definitions sensitive enough to detect majority of cases in the community Yes No

89. If yes, how? _________________________

90. If no, how? ________________________

91. How many false positive cases were reported in your system? --------------------

PRIVATE CLINIC/HOSPITALS LEVEL SURVEILLANCE SYSTEM EVALUATION QUESTIONNAIRE

I. IDENTIFIERS

Assessor team----------------------- Type of health facility ---------------

Date …………. District ----------------

Interviewer ------------------- City administration/province ----------------

Respondent ----------------- Name of health facility ------------------

II. SURVEILLANCE SYSTEM

1. Percent of health facilities with national surveillance manual

Is there a national manual for surveillance at this site? [Obs] Observed national surveillance manual:

Yes, No Unknown Not applicable

2. If yes list what type of manual -----------------------------

3. If no why? -------------------

III. CASE DETECTION AND REGISTRATION _______________

4. Percent of HFs that have a clinical register [Obs] Observed the existence of a clinical register

Yes, No Unknown Not applicable

5. Percent of health facilities that correctly register cases

[Obs] Observed the correct filling of the clinical register during the previous 30 days

Yes, No Unknown Not applicable

6. Percent of health facilities that have standardized case definitions for the country's priority diseases. Do you have a standard case definition for: (each priority disease) AWD, AFP (polio), measles, malaria, ----------------, --------------?

Yes No Unknown Not applicable

7. If not yes, describe ----------------------------

8. [Obs] Observed the standard case definition for: (each priority disease)

Yes, No Unknown Not applicable

9. Percent of health facilities that use standardized case definitions for the country's priority diseases

Obs Observed the respondent correctly diagnosing one of the country's priority diseases using a standard case definition

Yes, No Unknown Not applicable

10. Percentage of case definitions understood by professionals working at case identification site? ------------------------

(Select one of the priority diseases in the facility's clinical registers and asks how they diagnosed it — interviewer should have the standard case definition from MOH)

IV. CASE CONFIRMATION __________________

11. *P*ercent of health facilities that have the capacity to collect specimens (sputum stool, blood/serum and CSF). At this facility are you able to collect

Sputum	Y	N	U	N/A
Stool	Y	N	U	N/A
Blood	Y	N	U	N/A
CSF	Y	N	U	N/A

12. If not yes to any of the above please describe it & what alternative they have? ------------------ -------------------------

13. Observed the presence of materials required to collect

Stool	Y	N	U	N/A
Blood / serum	Y	N	U	N/A
CSF	Y	N	U	N/A
Sputum	Y	N	U	N/A

14. If not yes to any of the above please describe it & what alternative they have? ------------------ -------------------------

15. Percent of health facilities that have the capacity to handle specimens until shipment

Do you have the capacity to handle sputum, stool, blood/serum and CSF until shipment at this facility? Yes, No Unknown Not applicable

16. Observed presence of functional cold chain at health facility

Yes, No Unknown Not applicable

17. Percent of health facilities that have the capacity to ship specimens to higher level laboratories. Observed presence of transport media for stool at health facility

Yes, No Unknown Not applicable

18. [Obs]Observed presence of packing materials for shipment of specimens at health facility

Yes No Unknown Not applicable

V. DATA REPORTING ____________________________________

Percent of sites that have appropriate surveillance forms for that site at all times over the past 6 months

19. Have you lacked appropriate surveillance forms at any time during the last 6 months?

Yes, No Unknown Not applicable

20. If other than yes, describe --

21. Was there any report of the immediately reportable diseases in the past 1 month?

Yes No

22. If yes, what amount of time it was/were required to detect the case?

1. <24hr 2. <72hr 3. < a week 4. < a month 5. Specify if other

23. If yes to Q12, with in what time is the case notified after detection to the next level?

1. < one hour 2. (2-24) hrs. 3. (1- 2) days 4. (3- 7) days 5. After one week

24. Percent of sites that reported accurately cases from the registry into the summary report to go to higher level

[Obs] Observed that the last monthly report agreed with the register for 6 diseases (1 for each targeted group [eradication; elimination; epidemic prone; major public health importance])

Obs Measles	Y	N	U	N/A
Obs Malaria	Y	N	U	N/A
Obs AFP (polio)	Y	N	U	N/A
Obs AWD	Y	N	U	N/A
Obs -----------------	Y	N	U	N/A
Obs -------------------	Y	N	U	N/A

25. Percent of sites that reported each reporting period to the next higher level during the past 3 months

Number of reports in the last 3 months compared to expected number

Obs Weekly: /12 times the number of sites

Obs immediately: /-- times the number of sites

26. On time (use national deadlines)

[Obs] Number of weekly reports submitted on time:-_____ /12 times the number of sites

[Obs] Number of immediately reports submitted on time: ___/-- times the number of sites

27. Percent of HF that have means for reporting to next level by e-mail, telephone, fax or radio How do you report?

Mail   Fax   Telephone   Radio

Electronic mail   Other (specify)

28. Strengthening reporting How can reporting be improved?

VI. DATA ANALYSIS ____

Percent of sites that:

29. Describe data by person (Case-based, outbreaks) [Obs] Observed description of data by age and sex.   Yes,   No   Unknown   Not applicable
30. Describe data by place [Obs] Observed description of data by place (locality, village, work site. etc.)   Yes,   No   Unknown   Not applicable
31. Describe data by time [Obs] Observed description of data by time

    Yes,   No   Unknown   Not applicable 32. 32.
32. Perform trend analysis [Obs] Observed line graph of cases by time

    Yes,   No   Unknown   Not applicable
33. Describe their view if not yes for any of the three (case based, outbreaks, sentinel) -------------

    ----
34. Have an action threshold for each priority disease? Do you have an action threshold for any of the Country priority diseases?

    Yes   No   Unknown   Not applicable
35. If yes, what is it (Ask for 2 priority diseases)? ________cases ____ % increase _____rate
36. Please describe if answer to Q#41 is other than yes, -----------------------------------
37. Who is responsible for data analysis? ___________________________
38. How often do you analyze the collected data?

    Daily   Weekly   Every2 weeks   Monthly   Quarterly   As needed

XIII. OUTBREAK INVESTIGATION

39. Have you participated in any outbreak investigation?

    Yes   No
40. Where when & on what? ---------------------------------

41. Was/were there suspected outbreak detected in your H/Facility?

Yes No

42. Number of outbreaks suspected in the past year: ______________________

43. List the diseases: ____

XIV. EPIDEMIC PREPAREDNESS___________

44. Percent of health facilities that have a standard case management protocol for epidemic prone diseases, observed the existence of a written case management protocol for 1 epidemic prone disease. Yes, No Unknown Not applicable

45. Have you participated in any of epidemic management committee or RRT?

Yes, No Unknown Not applicable

46. If yes, describe where & when? ------------------

47. If not, why do you think? -------------------------------------

VII. EPIDEMIC RESPONSE___________________________________

Percent of sites that implemented prevention and control measures based on local data for at least one epidemic prone disease

48. Has the health facility implemented prevention and control measures based on local data for at least one epidemic prone disease?

Yes, No Unknown Not applicable

49. If not yes, discuss it -----------------

50. If yes how, _____________

VIII. FEEDBACK ____

Percent of sites that have received a report or bulletin from a higher level during the past year on the data they have provided

51. How many feedback bulletin or reports has the health facility received in the last year? ___

[Obs] Observed at least 1 report or bulletin at the health facility from a higher level during the past year on the data they have provided

Yes, No Unknown Not applicable

IX. SUPERVISION: ____

Percent of individuals supervised in the past 6 months

52. How many times have you been supervised in the last 6 months? _________

[Obs] Observed supervision report or any evidence of supervision in last 6 months

Yes, No Unknown Not applicable

53. Of those supervised in the previous 6 months, percent of individuals for which the supervisor from the next higher level reviewed surveillance practices appropriate to their level

[Obs] Observed supervision report or any evidence for appropriate review of surveillance practices

Yes, No Unknown Not applicable

X. TRAINING _______________________________________

54. Percent of health personnel trained in disease surveillance and epidemic management
Have you been trained in disease surveillance and epidemic management?

Yes, No Unknown Not applicable

55. If yes, specify when, where, how long, by whom?

____

____

IX. RESOURCES_____________________________________

Percent of sites that have:

56. Logistics

Electricity Bicycles Motor cycles Vehicles

57. Data management

Stationery Calculator Computer Printer Statistical package

58. Communications

Telephone Fax Radio call Computers that have modems

59. Information education and communication materials

Posters Megaphone Other (Specify)----------------

60. Hygiene and sanitation materials

A, Spray pump B, Disinfectant

70. Protection materials (list)

_______________________________ _________________________

__________________________ _______________________________

__________________________ _______________________________

SATISFACTION WITH SURVEILLANCE SYSTEM

71. Are you satisfied with the surveillance system?

Yes, No Unknown Not applicable

72. If no, how can the surveillance system be improved?

____

____

____

73. What opportunities are there for integration of surveillance activities and functions (core activities, training, supervision, guidelines, resources etc.?)

____

____

____

PART TWO

QUESTIONNAIRE FOR ATTRIBUTES & LEVEL OF USEFULNESS:

I. LEVEL OF USEFULNESS OF THE SURVEILLANCE SYSTEM FOR THESE SELECTED PRIORITY DISEASES

1. Does the surveillance system help to detect outbreaks of these selected priority diseases early?

   Yes No

2. If yes, how? --------------------------------
3. If no, why? --------------------------------
4. Does the surveillance system help to estimate the magnitude of morbidity and mortality related to these diseases, including identification of factors associated with these diseases?

   Yes No

5. Does the surveillance system help to permit assessment of the effect of prevention and control programs? Yes No
6. Observe (confirmation): interventions and diseases trends analyzed

   Available Not available

7. Does the system stimulate any research related activities in the area?

   Yes No

8. If yes describe it ----------------------------

DESCRIBE EACH SYSTEM ATTRIBUTES:

A. SIMPLICITY:

9. Is the case definition of AWD, malaria, AFP (polio), _________, __________ and measles easy for case detection by all level health professionals? Yes No
10. If yes how? -------------------------
11. If no, why? ----------------------------
12. What are the organizations which need to receive reports of the surveillance data? ------------------------
13. Do you feel that additional data collected on a case are time consuming?

    Yes No

14. How long it takes to fill the format? <5 minute 10-15minuts >15 minutes

15. How long does it take to have laboratory confirmation of

AWD---------, Measles ----------, AFP --------, Malaria -------

16. Is it difficult to run and coordinate the surveillance system in your area?

Yes, No

17. If yes, how? --------------------------------
18. If no, why? -------------------------------
19. Are instructions and guidelines for identifying cases, completing surveillance formats simple

Yes, No

B. FLEXIBILITY:

20. Can the current reporting formats be used for other newly occurring health event (disease) without much difficulty? Yes No
21. If yes, how? ------------------------
22. If no, why? ---------------------------------
23. Do you think that any change in the existing procedure of case detection, reporting, and formats will be difficult to implement? Yes No

C. DATA QUALITY: ____________

(Completeness of the reporting forms/and validity of the recorded data)

24. Are the data collection formats for these priority diseases clear and easy to fill for all the data collectors/ reporting sites? Yes No
25. Observe: Review the last months report of these diseases Average number of *unknown or blank responses* to variables in each of the reported forms ____________________________
26. Percent of reports which are complete (that is with no blank or unknown responses) from the total reports
27. Reasons for not filling them ______________________

D. ACCEPTABILITY: _________________

28. Do you think all the reporting units accept and well engaged to the surveillance activities?

Yes No

29. If no, what is the reason for their poor participation in the surveillance activity?

A/ Lack of understanding of the relevance of the data to be collected

B/ No feedback / or recognition given by the higher bodies for their contribution; i.e. no dissemination of the analysis data back to reporting facilities

C/ Reporting formats are difficult to understand

D/ Report formats are time consuming

E/ Other: ____

30. Do surveillance personnel admit their contributions and inputs to the existing _ IDSR system were considered valuable? Yes No
31. Do surveillance respondents demonstrated satisfaction with their involvement and agree for its PH importance? Yes No
32. If no why? ________________________

E. REPRESENTATIVENESS:

33. Do you think, the populations under surveillance have good health seeking behavior for these diseases? Yes No
34. If yes how? ------------------------
35. If no, why? ---------------------------------------
36. Who do you think is well represented by the surveillance data?

    Urban Rural Both None
37. Why? _________________________

F. STABILITY:

38. Do you think your facility surveillance system has the ability to collect, manage, and provide data properly without failure in the future? Yes No
39. If yes, how? ______________________
40. If no, how? _________________________
41. Do you store surveillance data? Yes No
42. Do your data storage system safe & efficient? Yes No
43. If yes how? ____________________________
44. If no, why? _________________________
45. Do you have surveillance trained staff? Yes No

G. TIMELINESS: ------------------

46. Does the clinic report surveillance cases on time? *Yes* *No*

47. If no, why? ___________________________

48. Percent of units report on time ---------------------

49. What time was required to detect cases of any of your previous suspect cases ----------

50. What time was required to notify the detected event to the immediate next level -----

51. What time was required to start response to the detected event ___________

H. COMPLETENESS:

52. Were the unit report cases including late report(report of each week in 2022)?

Yes No

53. If no, why? ______________

54. Do you think the reported cases reflect occurrence and distribution of all cases in the population under surveillance Yes No

55. If yes, how? ________________________

56. If no why? _______________________

57. Is the system applicable for any of the sites (urban or rural) or other socio demographic too and the system draws reports from the lowest (community 1-5 networks) level to the upper

Yes No

I. PREDICTIVE VALUE POSITIVE

58. what proportion of cases identified by your definition were confirmed as case by the standard confirmatory test for any outbreaks in your area?

J. SENSITIVITY

59. Does your surveillance case-definitions sensitive enough to detect majority of cases in the community Yes No

60. If yes, how? _______________________

61. If no, how? ______________________

62. How many false positive cases were reported in your system? --------------------

SURVEILLANCE SYSTEM EVALUATION HEALTH POST LEVEL QUESTIONNAIRE

I. IDENTIFIERS

Assessor team----------------------- Type of Health post/community structure---------------

Date …………. District ----------------

Interviewer ------------------- City administration/province ----------------

Respondent ----------------- Name of health Post; ------------------

XI. SURVEILLANCE SYSTEM

1. Percent of health HP with surveillance manual

Is there a surveillance manual for surveillance at this site?

[Obs] Observed national surveillance manual:

Yes No Unknown Not applicable

2. *If yes,* describe (last update, diseases included, case definitions, surveillance and control, integrated or different for each disease): ____ ____ ____ ____

3. If no why? ----------------------

XII. CASE DETECTION AND REGISTRATION ________________

1. Percent of Health post that have a case register/ rumor logbook

[Obs] Observed the existence of a case register/logbook

Yes No Unknown Not applicable

2. Percent of Hp that correctly register cases

[Obs] Observed the correct filling of the case register/logbook during the previous 30 days

Yes No Unknown Not applicable

3. Percent of Hp that have standardized community case definitions for the country's priority diseases

Do you have a standard community case definition for: (each priority disease) AWD, AFP (polio), measles, malaria?

Yes, No Unknown Not applicable

4. If not yes, describe -------------------------------
5. [Obs] Observed the standard community case definition for: (each priority disease)

   Yes, No Unknown Not applicable

6. Percent of HPs that use standardized community case definitions for the country's priority diseases

Obs Observed the respondent correctly diagnosing one of the country's priority diseases using a standard community case definition

(Select one of the priority diseases in the HPs case registers and asks how they diagnosed it — interviewer should have the standard community case definition from EPHI/District)

Yes, No Unknown Not applicable

7. Percentage of case definitions understood by professionals working at case identification site? -------------------------

XV. CASE CONFIRMATION ___________________

8. How could a suspected cases was/were confirmed in your area? ___________________ ___________ ________________________________

XIII. DATA REPORTING ___________________________________

Percent of sites that have appropriate surveillance forms for that site at all times over the past 6 months

9. Have you lacked appropriate surveillance forms at any time during the last 6 months?

   Yes, No Unknown Not applicable

10. Percent of sites that reported accurately cases from the case/rumor registry into the summary report to go to higher/ health center/ level

[Obs] Observed that the last monthly report agreed with the case/rumor/register for 6 diseases (1 for each targeted group [eradication; elimination; epidemic prone; major public health importance])

Obs Measles Y N U N/A

Obs Malaria Y N U N/A

Obs AFP (polio) Y N U N/A

Obs --------------------- Y N U N/A

Obs ------------- Y N U N/A

Obs ------------ Y N U N/A

11. Percent of HPs/sites/ that reported each reporting period to the next higher level during the past 3 months

Number of reports in the last 3 months compared to expected number

Obs Weekly: /12 times the number of sites

Obs immediately: /-- times the number of sites

12. On time (use national deadlines)

[Obs] Number of weekly reports submitted on time:-_____ /12 times the number of sites

[Obs] Number of immediately reports submitted on time: ___/-- times the number of sites

13. Was there any report of the immediately reportable diseases in the past 1 month?

Yes No

14. If yes, what amount of time it was/were required to detect the case?

1. <24hr 2. <72hr 3. < a week 4. < a month 5. Specify if other

15. If yes to Q12, with in what time is the report received after detection of the case/ diseases?

1. < one hour 2. (2-24) hrs. 3. (1- 2) days 4. (3- 7) days 5. After one week

16. Percent of HPs that have means for reporting to next level by e-mail, telephone, fax or radio How do you report?

Mail Telephone Radio

Electronic mail Other (specify)

17. Strengthening reporting How can reporting be improved?

DATA ANALYSIS ____

Percent of sites that:

18. Describe data by person (outbreaks, sentinel) [Obs] Observed description of data by age and sex. Yes, No Unknown Not applicable

19. Describe data by place [Obs] Observed description of data by place (locality, village, work site. Yes, No Unknown Not applicable

20. Describe data by time [Obs] Observed description of data by time

Yes, No Unknown Not applicable

115. Describe their view if not yes for any of the three (case based, outbreaks, sentinel) --------
----

116. Perform trend analysis [Obs] Observed simple line graph or bar graph of cases by time

Yes, No Unknown Not applicable

117. List disease(s) for which line graph is observed ___________________________________
____
____

118. Have an action threshold for each priority disease? Do you have an action threshold for any of the Country priority diseases?

Yes No Unknown Not applicable

119. If yes, what is it (Ask for 2 priority diseases)? _______cases ____ % increase _____rate

120. Please describe if answer to Q#29 is other than yes, ----------------------------------

121. Who is responsible for data analysis? __________________________

122. How often do you analyze the collected data?

Daily Weekly Every2 weeks Monthly Quarterly As needed

123. Have appropriate denominators Observed presence of demographic data at site (E.g. population <5yr., population by village, total population)

` Yes No Unknown Not applicable

XVI. OUTBREAK INVESTIGATION

124. Have you participated in outbreak investigation?

Yes No

125. Where when & on what? ---------------------------------

126. Was/were there suspected outbreak detected in your catchment?

Yes No

127. Number of outbreaks suspected in the past year: _______________________

128. List the diseases: ____

129. Of the investigated outbreaks in the past 1 year, percent in which risk factors were looked for:

130. Number of outbreaks in which risk factors were used for action: ______________

131. Percent in which findings were used for action [Observed report] -----------------------------
--

XVII. EPIDEMIC PREPAREDNESS___________

132. Percent of health posts that have a standard case management (follow up, referral e.c) protocol for epidemic prone diseases, observed the existence of a written document.

Yes, No Unknown Not applicable

133. Existence of a health facility plan for epidemic preparedness and response either alone or with the help of the health center. Observed a written plan of epidemic preparedness and response

Yes, No Unknown Not applicable

134. If not yes, describe it ---------------------------

135. Existence of emergency stocks of drugs, vaccines, and supplies at all times in past 1 year:

136. Has the HP had emergency stocks of drugs, vaccines, and supplies at all times in past 1 year? Yes, No Unknown Not applicable

137. If not yes, describe it ---------------------------

138. Experience of a shortage of drugs, vaccines or supplies during the most recent epidemic (or outbreak). Has the HP experienced shortage of drugs, vaccines or supplies during the most recent epidemic (or outbreak)?

Yes, No Unknown Not applicable

139. If not yes, describe it ---------------------------

140. Have you been a member of rapid response team for epidemics (RRT)?

Yes, No Unknown Not applicable

141. If yes, where, when? ----------------------------

XVIII. EPIDEMIC RESPONSE____________________________________

142. Percent of HPs/sites/ that implemented prevention and control measures based on local data for at least one epidemic prone disease either alone or in collaboration with health center. Has the HP implemented prevention and control measures based on local data for at least one epidemic prone disease?

Yes, No Unknown Not applicable

143. If yes, describe its best findings --------------------------------------

144. If not yes, why? ---

XIX. FEEDBACK ____

Percent of HPs/sites that have received a report or bulletin from a higher level/ woreda or district/ during the past year on the data they have provided

53. How many feedback bulletin or reports has the HPs received in the last year? ___

[Obs] Observed at least 1 report or bulletin at the HPs from a higher level during the past year on the data they have provided

Yes, No Unknown Not applicable

54. Percent of HPs that conducted at least semi-annual meetings with community members to discuss results of surveillance or investigation data

How many meetings has this HPs conducted with the community members in the past six months?

____

54. Observed the minutes or report of at least 1 meeting between the HPs team and the community members within the six months

Yes, No Unknown Not applicable

XX. SUPERVISION: ____

Percent of HPs supervised in the past 6 months

55. How many times have you been supervised in the last 6 months? __

[Obs] Observed supervision report or any evidence of supervision in last 6 months

Yes, No Unknown Not applicable

56. Of those supervised in the previous 6 months, percent of HPs for which the supervisor from the next higher level reviewed surveillance practices appropriate to their level

[Obs] Observed supervision report or any evidence for appropriate review of surveillance practices

Yes, No Unknown Not applicable

57. Do you have a plan for supporting the lower community health structures like CEBS local?

Yes, No Unknown Not applicable

145. If not yes, why? ----------------------------

146. How many supervisory visits have you made in the last 6 months? ____________________

147. Were your supervisions reasonably regular?

Yes, No Unknown Not applicable

148. The most usual reasons for not making all required supervisory visits. (Text)______________

____

____ _______________

XXI. TRAINING _____________________________________

58. Percent of health extension worker trained in community disease surveillance and epidemic management

Have you been trained in community-based disease surveillance and epidemic management?

Yes, No Unknown Not applicable

59. If yes, specify when, where, how long, by whom?

____

____

IX. RESOURCES___________________________________

Percent of sites that have:

60. Logistics: Electricity Bicycles Motor cycles

61. Data management: Stationery Printer Other (Specify)------

62. Communications Telephone Other(specify)--------

63. Information education and communication materials

Posters Megaphone Flipcharts or Image box. Other(specify) ------

64. Hygiene and sanitation materials ---------------------------------

65. Protection materials (list) ____

____________________________ ____________________________________

SATISFACTION WITH COMMUNITY SURVEILLANCE SYSTEM_______________

66. Satisfaction with the community surveillance system

Are you satisfied with the community surveillance system in your catchment?

Yes, No Unknown Not applicable

67. *If no*, how can the community surveillance system be improved?

____

____

68. What opportunities are there for integration of the community surveillance activities and functions (core activities, training, supervision, guidelines, resources etc.?)

____

____

____

PART TWO

QUESTIONNAIRE FOR ATTRIBUTES & LEVEL OF USEFULNESS:

1. Total population of the HPS catchment area under surveillance _____________
2. What is the case & death level of the commonest diseases in your catchment?

   -------------- cases __________ Deaths ________

   ---------------- cases __________ Deaths ________

   ________ cases __________ Deaths ________
3. Does the surveillance system help to detect outbreaks of these selected priority diseases early?

   Yes No
4. If yes, how? ---------------------------------
5. If no, why? ---------------------------------
6. Does the surveillance system help to estimate the magnitude of morbidity and mortality related to these diseases? Yes No
7. Does the surveillance system help to permit assessment of the effect of prevention and control programs? Yes No
8. Does the system help to identify priority conditions & most at risk conditions?

   Yes No
9. If yes how? --------------
10. If no why? ----------------------

DESCRIBE EACH SYSTEM ATTRIBUTES:

A. SIMPLICITY:

11. Is the community case definition of AWD, Rash, AFP (polio), easy for case detection?

    Yes No

12. If yes how? --------------------------
13. If no, why? -----------------------------
14. What are the organizations which need to receive reports of the surveillance data? ------------ -------------
15. Do you feel that additional data collected on a case are time consuming?

    Yes No

16. How long it takes to fill the format? <5-minute 10-15minuts >15 minutes
17. Is it difficult to run and coordinate the surveillance system in your area?

    Yes, No

18. If yes, how? ---------------------------------
19. If no, why? --------------------------------
20. Are instructions and guidelines for identifying cases, completing surveillance formats simple

    Yes, No

B. FLEXIBILITY:

21. Can the current reporting formats be used for other newly occurring health event (disease) without much difficulty? Yes No
22. If yes, how? -------------------------
23. If no, why? ------------------------------------
24. Do you think that any change in the existing procedure of case detection, reporting, and formats will be difficult to implement? Yes No
25. If yes how? ---------------------------
26. If no, why? __________________

27. Which do you think could possibly bring difficult problem in your surveillance system? Why?

New health-related events, Variations in funding

Changes in case definitions Reporting sources

Change in Technology

C. DATA QUALITY: ________________

(Completeness of the reporting forms/and validity of the recorded data)

28. Are the data collection formats for these priority diseases clear and easy to fill?

Yes No

29. If yes how? --------------------------

30. If no, why? _________________

31. Are the community surveillance focal persons (WDA) trained/ supervised regularly?

Yes No

32. If no, why? _____________________

33. Observe: Review the last months report of these diseases average number of *unknown or blank responses* to variables in each of the reported forms __________________________

34. Percent of reports which are complete (that is with no blank or unknown responses) from the total reports

---

D. ACCEPTABILITY:

35. Do you think all the reporting agents accept and well engaged to the surveillance activities?

Yes No

36. If yes, how many are active participants (of the expected to)? ______________

36. If no, what is the reason for their poor participation in the surveillance activity?

A/ Lack of understanding of the relevance of the data to be collected

B/ No feedback / or recognition given by the higher bodies for their contribution; i.e. no dissemination of the analysis data back to reporting facilities

C/ Reporting formats are difficult to understand

D/ Report formats are time consuming

E/ Other: ____

37. Do the end-users are willing to accept and use data generated through the system?

Yes No

38. If yes how? -------------------------------

39. If no, how? ----------------------------------

40. Do surveillance personnel admit their contributions and inputs to the existing _ IDSR system were considered valuable? Yes No

41. Do surveillance respondents demonstrated satisfaction with their involvement and agree for its PH importance? Yes No

42. If no why? ________________________

E. REPRESENTATIVENESS:

43. What is the health service coverage of the catchment? _________%

44. Do you think, the populations under surveillance have good health seeking behavior for these diseases? Yes No

45. If yes how? -----------------------

46. If no, why? -------------------------------------

47. Who do you think which is well represented by the surveillance data?

Urban Rural Both None

48. Why? _________________________

F. STABILITY:

49. Do you think your catchment surveillance system has the ability to collect, manage, and provide data properly without failure in the future? Yes No

50. Was there an experience of lack of resources that interrupt the surveillance system?

Yes No

51. If yes, how? ________________

52. Do you store data? Yes No

53. Do your data storage system safe & efficient? Yes No

54. If yes how? ___________________________

55. If no, why? ________________________

G. TIMELINESS: ------------------

56. Does the health posts report on time? *Yes* *No*

57. If no, why? ___________________________

58. Percent of units report on time --------------------------

59. What time was required to detect cases of any of your previous outbreaks ----------

60. What time was required to notify the detected event to the immediate next level -----

61. What time was required to start response to the detected event ___________

Weekly HP reports received on time for 2022 report by WHO epidemic week to be field at district health department

WHO week	Expected					Reported				
	HP	CEBS Focal	Others			HP	CEBS Focal	Others		

H. COMPLETENESS:

62. Are all the units report including late report? Yes No

63. If no, why? ______________

64. Percent of facilities that send report of each week in 2022

65. Do reported cases reflect occurrence and distribution of all cases in the population under surveillance Yes No

66. If yes, how? ________________________

67. If no why? ______________________

68. Is the system applicable for any of the sites (urban or rural) or other socio demographic too and the system draws reports from the lowest (community 1-5 networks) level to the upper Yes No

I. PREDICTIVE VALUE POSITIVE

69. The proportion of epidemics identified by the surveillance system that are true epidemics-----

70. what proportion of cases identified by your definition were confirmed as case by the standard confirmatory test for any outbreaks in your area?

J. SENSITIVITY

71. Does your surveillance case-definitions sensitive enough to detect majority of cases in the community Yes No
72. If yes, how? ________________________
73. If no, how? _______________________
74. How many false positive cases were reported in your system? ---------------------

SURVEILLANCE SYSTEM EVALUATION COMMUNITY SURVEILLANCE FOCAL LEVEL QUESTIONNAIRE

II. IDENTIFIERS

Assessor team----------------------- Date ………… District ----------------

Interviewer ------------------- Kebele, ----------------

Respondent ----------------- Name of community unit ------------------

XIV. SURVEILLANCE SYSTEM

1. Percent of health CSFs with surveillance material/leaf lates/Posters or any material

[Obs] Observed

Do you have any posters or case definitions to be used in your area

Yes No Unknown Not applicable

XV. CASE DETECTION _______________

2. Percent of community unit that have a case detection report --------------------

3. Percent of community structures that have standardized community/syndromic/case definitions for the country's priority diseases

Do you have a standard community case definition for: (each priority disease) Diarrheal disease AFP (polio), Rash, Febrile illness?

Yes No Unknown Not applicable

4. [Obs] Observed the standard community case definition for: (each priority disease)

Yes No Unknown Not applicable

5. Percent of community surveillance focal (CSF) that use standardized community case definitions for the country's priority diseases

Obs Observed the respondent correctly diagnosing one of the country's priority diseases using a community case definition

Yes No Unknown Not applicable

XVI. DATA REPORTING _____________________________________

6. Percent of sites that reported accurately cases from their suspect cross check it in HPs register -----------------

7. Percent of CSFs that reported each reporting period to the HP in the past 3 months

Number of reports in the last 3 months compared to expected number

Obs Weekly: /12 times the number of sites

8. On time (use national deadlines)

[Obs] Number of weekly reports submitted on time: _____ /12 times the number of sites

[Obs] Number of immediately reports submitted on time: ___/-- times the number of sites

9. Percent of CSFs that have means for reporting to next level by

____

10. Strengthening reporting, how can reporting be improved?

XVII. EPIDEMIC RESPONSE___________________________________

11. Has CSFs participated in prevention and control measures along with the HP?

Yes No Unknown Not applicable

XVIII. FEEDBACK ____

12. Percent of CSFs that have received a report or bulletin/information from a higher level/ HPs/ during the past year on the data they have provided ---------

13. Do you get feedback? Yes No Unknown Not applicable

How many feedback or reports has the CSF received in the last year? ___

14. Percent of CSFs that conducted at least semi-annual meetings with community members to discuss results of surveillance or investigation data -------------------

15. How many meetings has this HPs conducted with the community members in the past six months? _______________

XIX. SUPERVISION: ____

16. Percent of CSF supervised by HPs in the past 6 months ------------------

17. How many times have you been supervised in the last 6 months? __

XX. TRAINING _____________________________________

18. Percent of CSFs trained in community disease surveillance activities

Have you been trained in community-based disease surveillance

Yes No Unknown Not applicable

19. If yes, specify when, where, how long, by whom?

____

____

IX. RESOURCES________________________________

Percent of CSFs that have:

20. Logistics to be used for surveillance

What -------------

21. Data handling material ---------------------------------

22. Communications material _________________________

23. Information education and communication materials _____________________________

24. Hygiene and sanitation materials ______________________________

25. Protection materials (list)

____

X. SATISFACTION WITH COMMUNITY SURVEILLANCE SYSTEM______________

26. Satisfaction with the community surveillance system

Are you satisfied with the community surveillance system in your catchment?

Yes No Unknown Not applicable

27. *If no*, how can the community surveillance system be improved?

____

____

III. OPPORTUNITIES FOR INTEGRATION COMMUNITY SURVEILLANCE

1. What opportunities are there for integration of the community surveillance activities and functions (core activities, training, supervision, guidelines, resources etc.?)

____

____

____

PART TWO

QUESTIONNAIRE FOR ATTRIBUTES & LEVEL OF USEFULNESS:

1. Total population /House hold/ of the under your sites ____________

2. What is the suspected case & death happened in unit that you encountered?

-------------- cases __________ Deaths _________

---------------- cases __________ Deaths _________

_________ cases __________ Deaths _________

IV. LEVEL OF USEFULNESS OF THE SURVEILLANCE SYSTEM FOR THESE SELECTED PRIORITY DISEASES

1. Does the surveillance system help to detect cases of these selected priority diseases early?
   Yes No
2. Does the surveillance system help to permit assessment of the effect of prevention and control programs? Yes No

V. DESCRIBE EACH SYSTEM ATTRIBUTES:

a. SIMPLICITY:

Is the community case definition of AWD, Rash, AFP (polio), easy for case detection?

Yes No

1. What are the organizations which need to receive reports of the surveillance data from you? ------------------------
2. Do you feel that additional data collected on a case are time consuming?
   Yes No
3. How long it takes to see a suspected case? ----------------------------

b. FLEXIBILITY:

4. Can the current reporting system be used for other newly occurring health event (disease) without much difficulty? Yes No
5. Do you think that any change in the existing procedure of case detection & reporting, will be difficult to implement? Yes No

c. DATA QUALITY: (Completeness of the reporting forms/and validity of the recorded data)

6. Are the data required to be collected & reported to the health post for these priority diseases clear and easy? Yes No
7. Are the community members oriented visited regularly by you?
   Yes No

8. Percent of reports delivered by the CSFs which are complete (please check at the health post level for him) from the total reports
