# Supplementary material for: Evaluating the Integrated Disease Surveillance and Response system in Sidama Region, Ethiopia: A systems evaluation
Source: PLoS One. 2026 May 21;21(5):e0349856. doi: 10.1371/journal.pone.0349856 (PMC13193418; doi:10.1371/journal.pone.0349856)
Supplement: Attachment — Submitted filename: Point by point response.docx [file pone.0349856.s004.docx]

# Response to Reviewers

**Manuscript Title:** Evaluating the Integrated Disease Surveillance and Response System in Sidama Region, Ethiopia: A Systems Evaluation

**Manuscript ID:** PONE-D-25-41162

Dear Dr. Iezadi,

Thank you for the opportunity to revise our manuscript entitled "Evaluating the Integrated Disease Surveillance and Response System in Sidama Region, Ethiopia: A Systems Evaluation" (PONE-D-25-41162). We appreciate the constructive feedback provided by you and the reviewers. We have carefully addressed all comments in the revised manuscript and provide below a point-by-point response.

All changes to the manuscript are highlighted using track changes. We believe the revisions have substantially strengthened the methodological transparency, analytical rigor, and policy relevance of our work.

Respectfully submitted,

Sileshi Demelash Sasie (on behalf of all authors)

## Response to Reviewer #1

**General Comment:** The reviewer thanks the authors and states that the manuscript addresses a topic of practical importance for strengthening health surveillance systems in low- and middle-income countries.

**Response:** We thank the reviewer for the positive assessment of the manuscript's relevance and potential value from a health policy perspective. We have carefully addressed each methodological concern raised.

### **Comment 1.1: Sampling Strategy Justification**

**Comment:** The combination of random and purposive sampling without sufficient methodological justification may introduce selection bias and limit the generalizability of the results.

**Response:** We agree that transparency regarding sampling decisions is essential. We have substantially expanded the sampling justification to clarify that the multistage design employed different sampling strategies appropriate to each tier's evaluation objective: simple random sampling at the district level to support descriptive generalizability, and purposive sampling at lower tiers to focus on active surveillance actors.

**Revisions:** Because the objective was to evaluate the operational performance of the surveillance system among active surveillance actors rather than to estimate population prevalence, purposive selection of health posts and HDA members with known surveillance roles is methodologically appropriate for systems evaluations. Simple random sampling at the district level supports descriptive generalizability for district-level findings. However, we acknowledge that purposive sampling at lower tiers limits generalizability to non-participating facilities and community actors (see methods). **(Page 6-7, line lines 132-138)**

Additionally, in the **Limitations and Strength** section, we added:

The use of purposive sampling at lower levels limits generalizability beyond the selected facilities and community actors, and because we selected only facilities with documented surveillance activity, our findings likely overestimate true population-level performance. **(Limitation section, page 21 line 352-353).**

### **Comment 1.2: Absence of Comparative Statistical Tests**

**Comment:** The analysis relies solely on descriptive statistics and does not include comparative statistical tests to examine differences across levels... some inferences presented in Results and Discussion are not fully supported by statistical evidence.

**Response:** We acknowledge this concern and have explicitly justified the descriptive-only analytical approach. Given the evaluative (rather than inferential) study design, non-probability sampling at lower tiers, and the hierarchical clustering of observations within facilities, inferential statistics would be inappropriate and potentially misleading. We have made this rationale transparent.

**Revisions:** Performance thresholds were defined a priori using WHO IDSR guidelines [13] and Ethiopian PHEM standards. Using these sources, 'adequate' was defined as: training coverage ≥80% of personnel trained within the preceding 2 years; supervision ≥1 supervisory visit per quarter; availability of surveillance forms ≥90% of facilities with no stockout in the preceding 6 months; and case definition adherence ≥90% for formal facilities and ≥80% for community actors (adjusted for literacy levels). **(Methods section, page 9-10, line 174-178).**

**Revisions**: Fourth, inferential statistics were not employed a deliberate choice given the descriptive, evaluative design, non-probability sampling, and hierarchical clustering of observations. Instead, we provide descriptive estimates with benchmarked interpretations, which are appropriate for systems evaluations. **(Limitations and Strength section, page 20, line 341-344))**

### **Comment 1.3:** Operational Definition of Key Performance Indicators

**Comment:** Key performance indicators such as adherence to case definitions and data quality be operationally defined.

**Response:** We agree entirely. We have now explicitly defined all performance indicators using a priori thresholds derived from WHO IDSR technical guidelines and Ethiopian PHEM standards.

**Revisions**: Performance thresholds were defined a priori using WHO IDSR guidelines [13] and Ethiopian PHEM standards. Using these sources, 'adequate' was defined as: training coverage ≥80% of personnel trained within the preceding 2 years; supervision ≥1 supervisory visit per quarter; availability of surveillance forms ≥90% of facilities with no stockout in the preceding 6 months; and case definition adherence ≥90% for formal facilities and ≥80% for community actors (adjusted for literacy levels). Added (lines 274-278): **(Methods section, page 9-10, line 174-178).**

**Revisions:** These benchmarks are now referenced throughout, including in the Abstract, Results section (opening paragraph), Tables, and Conclusions.

### **Comment 1.4:** Validity and Reliability of Data Collection Instrument

**Comment:** The validity and reliability of the data collection instrument be reported more transparently.

**Response:** We have expanded the description of instrument validation procedures to include details of pilot testing, expert review, and cultural adaptation.

**Revisions**: To ensure contextual and linguistic appropriateness, the tool was pilot-tested in one district of Sidama Region. Terminology and formatting were refined to account for varied literacy levels, using expert review by ten public health professionals. Questions were revised to ensure clarity, comprehensiveness, and cultural relevance. Feedback from the pilot also supported calibration of the checklist to local workflows and WHO IDSR guidelines. **(Methods section, page 7, 149-153)**

### **Comment 1.5:** Deeper Analytical Perspective in Discussion

**Comment:** The Discussion would benefit from a deeper analytical perspective, including clearer comparisons with similar studies conducted in other settings, as well as exploration of structural and managerial factors underlying the identified weaknesses.

**Response:** We have substantially revised the Discussion to include: (1) cross-country comparative analysis with Tanzania, Kenya, India, and Iraq; (2) exploration of structural factors including the "cascade effect" and budget flow mechanisms; and (3) explicit discussion of the distinction between structural capacity gaps and performance gaps.

**Revisions:** The observed disparities likely reflect underlying structural and managerial factors rather than isolated individual performance gaps. District-level offices receive direct budget allocations for training and supervision from the regional health bureau, whereas health posts and HDA members rely on intermittent, often unfunded support from health centers. This cascaded model of capacity building, while efficient for centralized resource allocation, results in a gradual dilution of training quality and reduced frequency of supervision at lower tiers. The 89 percentage-point gap in case definition adherence between public facilities (96%) and HDA members (4.2%) illustrates this cascade effect. Similar patterns have been documented in Tanzania and India [20,33], suggesting that decentralized surveillance systems require explicit bottom-up reinforcement mechanisms. **(Discussion section, page 17-18, line 278-289)**

In addition,

This study revealed substantial disparities in IDSR implementation across healthcare tiers in Sidama Region. Training coverage declined sharply from district to peripheral levels, with only 11% of health posts and 12% of HDA members receiving relevant training, reflecting a clear cascade gap consistent with patterns reported elsewhere in East Africa. In Tanzania, Saleh et al. [32] documented a similar gradient, with training coverage declining from 78% at district level to 34% at dispensary level. Likewise, supervisory visits reached only 61% of health posts in Sidama, comparable to findings from Kenya, where Ng'etich et al. [30] reported that only 41% of health posts had received supervisory visits. Within Ethiopia, our findings align with Alemu et al. [34], who evaluated the Dangila district surveillance system and similarly identified training gaps (only 45% of health workers trained) and inconsistent supervision as major barriers. However, the present study extends these findings by quantifying the cascade gap specifically at the HDA level (4.2% case definition adherence), a community-tier actor not included in previous Ethiopian evaluations. In Iraq, Hamalaw et al. [35] found that more than 25% of outbreaks were not notified within mandated timeframes, consistent with the preparedness and reporting delays observed in Sidama. Taken together, these cross-country patterns suggest that the challenges identified are not unique to Ethiopia but reflect broader systemic weaknesses in decentralized surveillance systems across comparable low- and middle-income settings. **(Discussion section, page 19-20, line 318-134)**

### **Comment 1.6**: Actionable Policy Recommendations

**Comment:** Providing more actionable policy recommendations would substantially improve the manuscript.

**Response:** We have added a dedicated **Implications and Recommendations** section with specific, tier-tailored, actionable recommendations.

**Revisions:**  The following actionable recommendations emerge from the findings:

1. **Establish dedicated outbreak response budgets** at district level and ensure regular supply of surveillance forms at health posts.
2. **Redesign training programs** to reach peripheral levels, supported by simplified case definition job aids and quarterly supervisory visits.
3. **Institutionalize routine data quality audits** with feedback loops from regional to community levels, using predefined benchmarks as monitoring targets.

Strengthening IDSR requires tier-specific interventions that address cascaded weaknesses at peripheral levels rather than one-size-fits-all approaches. (**Implications and Recommendations section, page 21, line 356-364)**

### **Comment 1.7:** Inconsistencies and Repetition

**Comment:** Some inconsistencies between figures reported in the text and tables, repetition across sections, and excessive length in certain parts should be addressed.

**Response:** We have carefully reviewed the manuscript and corrected all identified inconsistencies. Specifically:

1. **Data Analysis percentages (Table 1):** Corrected district-level analysis by person from 73% to 85% (11/13 facilities)
2. **Feedback percentages (Table 1):** Corrected public facilities feedback from 70% to 63% (17/27 facilities)
3. **Removed duplicate paragraph** justifying Sidama Region selection in Background section
4. **Standardized terminology:** "Health Development Army (HDA)" used consistently throughout

## Response to Reviewer #2

**General Comment:** The reviewer states the manuscript is technically sound and methodologically appropriate, with strong alignment to WHO IDSR frameworks and inclusion of community-level actors as a major strength.

**Response:** We thank the reviewer for the thorough and constructive assessment. We have addressed each recommendation below.

### **Comment 2.1:** Distinguish Structural Capacity Gaps from Performance Gaps

**Comment:** More explicitly distinguish structural capacity gaps from performance or outcome gaps.

**Response:** We have revised the Discussion to explicitly distinguish between structural capacity (availability of plans, budgets, personnel, guidelines) and operational performance (adherence to case definitions, data quality, trend analysis).

**Revisions:** The observed disparities likely reflect underlying structural and managerial factors rather than isolated individual performance gaps. District-level offices receive direct budget allocations for training and supervision from the regional health bureau, whereas health posts and HDA members rely on intermittent, often unfunded support from health centers. This cascaded model of capacity building, while efficient for centralized resource allocation, results in a gradual dilution of training quality and reduced frequency of supervision at lower tiers. **(Discussion section, page 17-18, line 278-283)**

### **Comment 2.2:** Explicit Performance Thresholds to Avoid Overgeneralization

**Comment:** Avoid occasional overgeneralization (e.g., 'majority of sites performed inadequately') without defining explicit performance thresholds.

**Response:** We have removed all vague generalizations and replaced them with threshold-referenced statements throughout the manuscript.

**Revisions:** Only 11% of health posts and 12% of HDA members reported receiving relevant training. Thus, neither health posts nor HDA members met the predefined ≥80% training coverage benchmark. (**Abstract section, lines 41-42)**

Additionally, in result section,

Using the a priori performance thresholds defined in the Methods (≥90% for formal facilities, ≥80% for community actors), we evaluated adherence across all levels. (**Results section, page 9, line 194-196)**

### **Comment 2.3:** Explicit Justification for No Inferential Statistics

**Comment:** Explicitly stating why inferential statistics were not pursued (e.g., non-probability sampling at later stages, system evaluation intent rather than hypothesis testing).

**Response:** We have added explicit justification as requested.

**Revisions:** Fourth, inferential statistics were not employed a deliberate choice given the descriptive, evaluative design, non-probability sampling, and hierarchical clustering of observations. Instead, we provide descriptive estimates with benchmarked interpretations, which are appropriate for systems evaluations. **(Limitations and Strength section, page 20, line 341-344)**

### **Comment 2.4:** Confidence Intervals or Variability Measures

**Comment:** Reporting confidence intervals or variability measures for key proportions, where feasible.

**Response:** While we acknowledge this suggestion, we respectfully note that confidence intervals are typically associated with inferential statistical frameworks. Our study is explicitly descriptive and evaluative, designed to assess system performance against predefined benchmarks rather than to estimate population parameters or test hypotheses. The a priori thresholds (≥90% for formal facilities, ≥80% for community actors) provide the appropriate interpretive framework. Adding confidence intervals would imply a sampling-based inferential approach that does not align with our purposive sampling at lower tiers or our evaluative design. We have clarified this in the manuscript.

**Revisions:** Transparent reporting of performance benchmarks allows readers to interpret gaps without overreliance on statistical significance. **(Limitations and Strength section, page 20-21, line 349-351)**

### **Comment 2.5:** Facility-Level Clustering Consideration

**Comment:** Clarifying whether facility-level clustering was considered when summarizing results across tiers.

**Response:** Thank you for this important methodological question.

We fully recognize that facility-level clustering exists in our data (e.g., multiple health posts within the same district, multiple HDA members within the same health post, and multiple facility types within the same catchment area).

However, for the following reasons, clustering was not statistically adjusted for in our analysis:

1. **Descriptive and evaluative design:** Our study was designed to describe system performance against predefined benchmarks, not to test inferential hypotheses. Clustering adjustments are typically required for inferential statistics (e.g., regression models with cluster-robust standard errors), which we did not perform.
2. **Non-probability sampling at lower tiers:** Health posts and HDA members were purposively selected based on active surveillance roles. Statistical adjustments for clustering assume probability-based sampling, which was not feasible or appropriate for this systems evaluation.
3. **Transparent acknowledgment:** We have explicitly acknowledged hierarchical clustering in the Limitations section, stating that "inferential statistics were not employed a deliberate choice given the descriptive, evaluative design, non-probability sampling, and hierarchical clustering of observations."

### **Comment 2.6:** Data Availability Statement Compliance

**Comment:** Revise the data availability statement to better align with PLOS ONE expectations.

**Response:** We have revised the Data Availability Statement to be more specific and transparent.

**Revisions:** The data underlying this study are derived from a sub-national surveillance system evaluation conducted in the Sidama Region of Ethiopia and cannot be deposited in a public repository due to ethical and institutional restrictions. De-identified data may be requested from the corresponding author (Sileshi Demelash Sasie, sileyeshi21@gmail.com) or from the Yekatit-12 Hospital Medical College Ethical Review Board, subject to institutional approval. **(Declaration section, page 22, line 384-388**):

### **Comment 2.7:** Language, Terminology, and Formatting

**Comment:** Repetitive sentences, occasional inconsistencies in terminology, formatting issues in tables, minor grammatical errors.

**Response:** We have systematically addressed all language and formatting issues:

1. **Removed duplicate paragraph** justifying Sidama Region selection (Background section)
2. **Standardized terminology:** "Health Development Army (HDA)" throughout (was inconsistently "health developmental army")
3. **Corrected table formatting:** All tables now have proper alignment and consistent percentage formatting
4. **Fixed typographical errors:** Corrected "common mmon deficiencies" to "common deficiencies"; "covera" to "coverage"
5. **Standardized reference format:** All references now follow consistent PLOS ONE style

### **Comment 2.8:** Integration of Results and Discussion

**Comment:** Some results (e.g., data analysis capacity, flexibility) are repeated in the Discussion without deeper synthesis.

**Response:** We have revised the Discussion to reduce redundancy and add analytical synthesis rather than mere restatement of results.

**Revisions:** Key attributes such as flexibility were positively regarded across most levels, but gaps persisted in data quality and monitoring sensitivity. Similar issues with data quality, analysis, and presentation have been identified in studies conducted in Tanzania and Iraq [20,22]. Poor data recording and reporting from clinics carrying out early detection threatens accurate regional surveillance and delays evidence-based outbreak responses. The very low adherence to case definitions among HDA members (4.2%) reflects not only training gaps but also the fundamental mismatch between clinically oriented case definitions and the practical, symptom-based surveillance feasible at the community level. **(Discussion section, page 19, line 309-319).**

## Summary of Major Revisions

| **Section** | **Key Changes** |
| --- | --- |
| **Abstract** | Added performance benchmarks and threshold-referenced interpretations |
| **Methods - Sampling** | Expanded justification for mixed sampling strategy |
| **Methods - Data Analysis** | Added a priori performance thresholds (WHO IDSR + Ethiopian PHEM) |
| **Methods - Instrument** | Expanded validity/reliability reporting (pilot test, expert review) |
| **Methods - Ethics** | Moved full ethics statement to Methods (2 paragraphs) |
| **Results** | Added benchmark-referenced reporting; corrected inconsistencies |
| **Discussion** | Added structural cascade analysis; cross-country synthesis; distinction between structural and performance gaps |
| **Limitations** | Added acknowledgment of purposive sampling limitations, hierarchical clustering, descriptive-only design |
| **Implications** | Added tier-specific actionable recommendations |
| **Declaration** | Removed redundant ethics statement; revised Data Availability Statement |
| **Tables** | Corrected formatting and percentages |
| **Language** | Standardized terminology; corrected typographical errors; removed repetition |

We believe the revised manuscript now meets the highest standards of methodological transparency and scientific rigor expected by PLOS ONE. We appreciate the reviewers' time and expertise, which have substantially improved this work.

We look forward to your favorable decision.

**Sincerely,**

Dr Sileshi Demelash Sasie (Corresponding Author)

On behalf of all co-authors
